# AGAMOUS Controls *GIANT KILLER*, a Multifunctional Chromatin Modifier in Reproductive Organ Patterning and Differentiation

**DOI:** 10.1371/journal.pbio.1000251

**Published:** 2009-11-24

**Authors:** Kian-Hong Ng, Hao Yu, Toshiro Ito

**Affiliations:** 1 Temasek Life Sciences Laboratory, National University of Singapore, Singapore; 2 Department of Biological Sciences, Faculty of Science, National University of Singapore, Singapore; 3 PRESTO, Japan Science and Technology Agency, Saitama, Japan; Max Planck Institute for Developmental Biology, Germany

## Abstract

The *Arabidopsis* homeotic protein AGAMOUS (AG), a MADS domain transcription factor, specifies reproductive organ identity during flower development. Using a binding assay and expression analysis, we identified a direct target of AG, *GIANT KILLER* (*GIK*), which fine-tunes the expression of multiple genes downstream of AG. The GIK protein contains an AT-hook DNA binding motif that is widely found in chromosomal proteins and that binds to nuclear matrix attachment regions of DNA elements. Overexpression and loss of function of *GIK* cause wide-ranging defects in patterning and differentiation of reproductive organs. GIK directly regulates the expression of several key transcriptional regulators, including *ETTIN/AUXIN RESPONSE FACTOR 3* (*ETT/ARF3*) that patterns the gynoecium, by binding to the matrix attachment regions of target promoters. Overexpression of *GIK* causes a swift and dynamic change in repressive histone modification in the *ETT* promoter. We propose that GIK acts as a molecular node downstream of the homeotic protein AG, regulating patterning and differentiation of reproductive organs through chromatin organization.

## Abstract 2

The floral, homeotic protein AGAMOUS coordinates multiple downstream genes through direct transcriptional regulation of the nuclear matrix attachment region binding protein *GIANT KILLER*.

## Author Summary

Multicellular development depends on proper expression of thousands of genes. Master regulators, such as homeotic proteins, code for transcription factors in both plants and animals and are thought to act by regulating other genes. Recent genomic studies in the plant Arabidopsis have shown that over 1,000 genes are regulated by homeotic proteins that directly control various target genes, including different classes of transcriptional regulators. It is not known, however, how expression of so many genes is coordinated by a single homeotic gene to form functional organs and tissues. Here we identified a transcriptional target of the plant homeotic protein AGAMOUS using bioinformatics analysis and showed that AGAMOUS directly controls GIANT KILLER, a multifunctional chromatin modifier. GIANT KILLER then binds to the upstream regions of multiple genes involved in patterning and differentiation in the AGAMOUS pathway and fine-tunes the expression of these genes. These data therefore provide a possible mechanism by which a homeotic gene coordinates multiple downstream targets in plants.

## Introduction

During flower development, many key processes depend on tissue-specific regulation of gene expression achieved by the coordinated interplay of transcription factors. The classical ABC model was proposed nearly two decades ago to account for organ identity control in flower development [1]. The ABC model predicts that the combinatorial action of ABC floral homeotic genes controls floral organ identity. The ABC genes, A class for *APETALA1* (*AP1*) and *APETALA2* (*AP2*), B class for *APETALA3* (*AP3*) and *PISTILLATA* (*PI*), and C class for *AGAMOUS* (*AG*), have been extensively studied and have been shown to encode transcription factors [2]–[8]. *AG* encodes a transcription factor of the MADS-domain protein family, and *AG* is necessary for the specification of stamens and carpels, the floral reproductive organs [5],[6]. In *ag-1* mutants, flowers undergo homeotic conversion to show a sepal-petal-petal reiteration instead of the normal sepal-petal-stamen-carpel structure. The complete lack of reproductive organs in *ag-1* flowers places *AG* at the top of the hierarchy of genes controlling reproductive development. This conclusion is supported by microarray expression profiling of wild-type and *ag* mutant flowers showing that more than 1,000 genes are regulated downstream of AG [9].

Genome-wide studies by microarray using plant lines with controllable floral homeotic activities and chromatin immunoprecipitation (ChIP) led to the identification of direct target genes of the homeotic proteins [10]–[14]. AG directly regulates *SPOROCYTELESS* (*SPL*, *NOZZLE*) [15],[16] to induce microsporogenesis, a process leading to pollen formation in *Arabidopsis* [12]. AG is expressed in developing stamens and regulates the expression of the catalytic enzyme DEFECTIVE IN ANTHER DEHISCENCE 1 (DAD1) [17] to induce the biosynthesis of the phytohormone jasmonate, which is required for stamen maturation [18].

Along with *SPL* and *DAD1*, genetic studies in *Arabidopsis* have revealed a large group of genes that are necessary for proper patterning and differentiation of reproductive organs. *ETTIN* (*ETT*, *AUXIN RESPONSE FACTOR3*) acts redundantly with *AUXIN RESPONSE FACTOR 4* (*ARF4*) to participate in abaxial-adaxial axis patterning of the floral meristem and reproductive organs, as well as in the apical-basal patterning of the gynoecium [19]–[21]. LEUNIG (LUG) is implicated as a negative regulator of *AG* in petal primordia and also controls gynoecium fusion [22]–[24]. The *YABBY* family gene *CRABS CLAW* (*CRC*) is expressed preferentially in the abaxial side of carpels and is involved in specification of the gynoecium and nectaries [25],[26]. *JAGGED* (*JAG*) and *NUBBIN* (*NUB*), both encoding C2H2 zinc-finger transcription factors, function redundantly to promote proliferation of stamen and carpel primordia [27]–[29]. Another C2H2 zinc-finger transcription factor, KNUCKLES (KNU), is involved in floral meristem determinacy and gametophyte specification, and its meristem expression is directly regulated by AG [30],[31]. Nevertheless, the genetic pathways and networks leading to organogenesis are largely unknown, as are the molecular mechanisms that orchestrate the large number of transcriptional gene circuits downstream of AG.

We report here the identification of *GIANT KILLER* (*GIK*), a gene coding for an AT-hook type DNA binding protein, as a target of AG. GIK belongs to a protein family consisting of 29 members in *Arabidopsis* [32],[33]. AT-hook DNA binding proteins may contribute to functional nuclear architecture by binding to the nuclear matrix [34]–[36]. The nuclear matrix is a putative structural component that remains inside the nucleus after removal of basic proteins and histones. AT-hook motifs bind to the minor grooves in duplex DNA of matrix attachment regions (MARs) of target DNA sequences [37],[38], a property that distinguishes them from common transcription factors that primarily bind to the major groove. MARs are stretches of characteristic AT-rich DNA sequences that tend to attain a single-stranded conformation through base unpairing (thus, MARs are also called base unpairing regions, or BURs) as a result of the torsional stress of the surrounding DNA [39]. MARs and AT-hook DNA binding proteins are believed to mediate anchoring of specific DNA sequences to the nuclear matrix, generating chromatin loop domains and possibly introducing structural changes in the chromatin [37]. In animals, the MAR binding protein SATB1, which contains an AT-hook motif, has been implicated in tissue- or cell-type-specific regulation of multiple genes [40]–[44]. SATB1 may play a role in chromatin assembly and histone modification of nearby genes and may influence the transcription of multiple target genes. In plants, very little is known about developmental roles of AT-hook motif proteins, although close homologs of *GIK* have been isolated using yeast one-hybrid screening as promoter-binding proteins as well as from activation tagging screens [33],[45]–[49].

We propose that *GIK* acts as a target of the floral homeotic protein AG and fine-tunes the expression of multiple genes involved in organ patterning and differentiation during reproductive development. Therefore, these data reveal one of the mechanisms by which homeotic genes regulate multiple downstream targets in plants.

## Results

### *GIK* Is a Direct Target of AG

**Figure 1:**
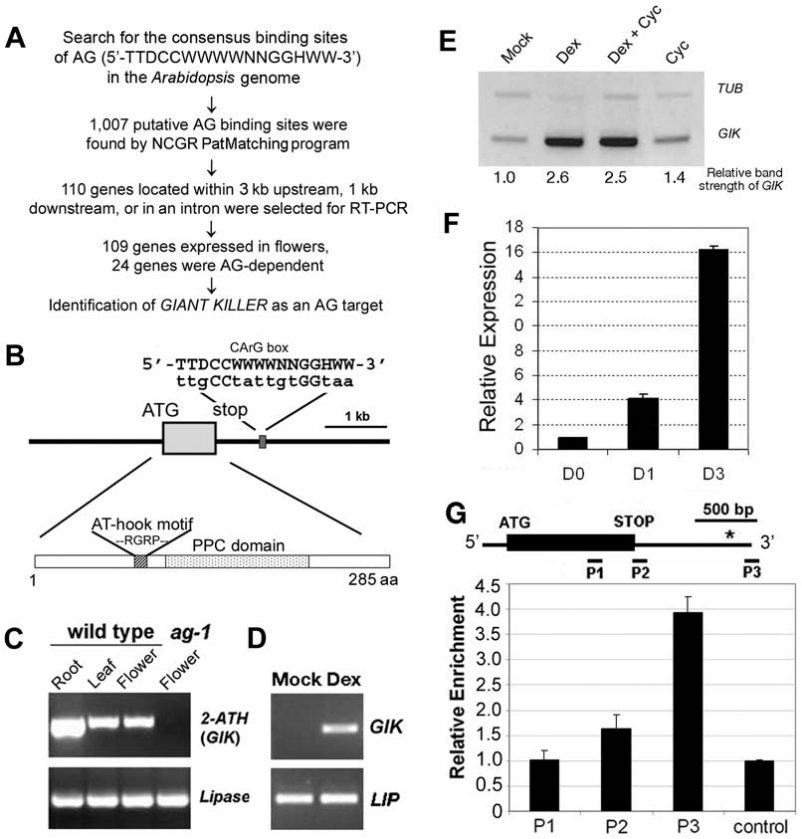
Identification of *GIK* as a direct target of AG. (A) Flow chart of the bioinformatic screening process. D = A/T/G; H = A/T/C; N = A/T/G/C, W = A/T. (B) Schematic diagram of the *GIK* genomic sequence and the predicted protein structure of GIK. The residues Arg (R)-Gly (G)-Arg (R)-Proline (P) are the AT-hook motif core. PPC = plant-and-prokaryote conserved domain. (C) Semi-quantitative RT-PCR analysis of *GIK* in wild-type tissues and *ag* mutant flowers. Shown at the bottom is the lipase that was amplified as a control. (D) Induction of *GIK* by AG in the inflorescences of *ag-1 35S::AG-GR* plants. Plants were mock- or DEX-treated and harvested 6 h after the treatment. Semi-quantitative RT-PCR was performed for *GIK* and *LIPASE* (*LIP*). (E) Induction of *GIK* by AG in the presence of a protein synthesis inhibitor. Inflorescences of *ag-1 35S::AG-GR* plants were mock-treated or treated with DEX, DEX plus cycloheximide (Cyc), or Cyc-only and harvested 2 h after the treatment. Semi-quantitative RT-PCR was performed for *GIK* and *TUBULIN2* (*TUB*). Each band strength was measured using ImageJ (http://rsb.info.nih.gov/ij/), and the relative band strength was calculated from the intensity of *GIK* normalized to that of *TUB*. (F) Real-time PCR analysis of *GIK* induction by AG. Inflorescences from *ag-1 AG-GR* plants were harvested 1 (D1) and 3 (D3) d after a single DEX treatment at day 0 (D0). Expression levels were normalized to that of *TUB*. The expression at D0 was set as 1.0. (G) AG binds to the *GIK* CArG box in vivo. ChIP was performed using *ag-1 35S::AG-GR* inflorescences at day 7 after four DEX treatments. P1, P2, and P3 indicate primer pairs used to detect different regions of *GIK* genomic DNA. The asterisk shows the location of the CArG box sequences. Relative enrichment was obtained from the ratio of enrichment achieved by anti-AG to that of control IgG. Enrichment of a sequence amplified from *PFK* genomic DNA was used as a basal control and was set to 1.0. Standard deviation was obtained from PCR triplicates.

We identified *At2G35270* (isolation name, *2-ATH*; *AHL21* [32]) as a putative direct target of AG using bioinformatics screening (Figure 1A, B) of the *Arabidopsis* genome for potential AG binding sites and named it *GIK* (as we found that it functions as a negative regulator of a gene whose name means “giant”; see below). First, we searched the entire *Arabidopsis* genome for the 16-bp consensus CArG box binding sequences of AG (5′-TTDCCWWWWNNGGHWW-3′, D = A/T/G, W = A/T, N = A/T/G/C, H = A/T/C) [50],[51] and found 1,007 sites (allowing one mismatch) by utilizing the NCGR Patmatch program (http://www.arabidopsis.org/cgi-bin/patmatch/nph-patmatch.pl). We then identified 110 genes located near the putative binding sites of AG (within 3 kb upstream, 1 kb downstream, or in introns) and tested their expression in wild-type and *ag* mutant flowers using RT-PCR (Figure 1A, Figure S1). Most of these genes were expressed in flowers. By comparing RNA in wild-type and *ag-1* mutant flowers, we found that 24 of these genes (22%) showed AG-dependent expression patterns (Figure S1, Table S1); of these, *GIK*, *SHATTERPROOF2* (*SHP2*, *AGL5*, At2g42830) [52],[53], and *ATHB40* (At4g36740) [54],[55] showed rapid induction upon AG activation in an inducible AG activity line [12] (unpublished data for *ATHB40*, see below for *GIK*). We found a typical CArG box sequence located 732 bp downstream of the translational termination codon of *GIK* (Figure 1B). After the series of experiments described below, we identified *GIK* as a direct target of AG. *GIK* encodes an AT-hook type DNA binding protein with an uncharacterized plant-and-prokaryote conserved domain (Figure 1B).

*GIK* transcripts were detected in roots, flowers, and leaves, with the highest expression in the roots, showing that *GIK* does not code for a flower-specific transcript (Figure 1C). In *ag* mutant flowers, *GIK* expression was substantially reduced (Figure 1C), suggesting that AG may be an upstream activator of *GIK* or that *GIK* is expressed in stamens and/or carpels, which are missing in *ag-1* mutant flowers. To clarify these two possibilities, we used *ag-1* plants that are transgenic for *35S::AG-GR* as a post-translational AG activation system [12] and analyzed the expression of *GIK* following AG induction in developing flowers by RT-PCR and real-time PCR with *GIK*-specific primer sets (Figure 1D–F; Table S2 for primer sequences). The transgenic line contains a gene coding for a fusion protein (AG-GR) of AG and the steroid binding domain of the rat glucocorticoid receptor (GR) on the *ag-1* mutant background. Following application of the synthetic glucocorticoid dexamethasone (DEX), AG-GR enters the nucleus and induces AG activity. *GIK* expression was upregulated 6 h after 10 µM DEX treatment compared to mock-treated inflorescences (Figure 1D). The induction was observed even 2 h after DEX treatment (Figure 1E). To exclude the possibility that *GIK* was induced indirectly by AG through an intermediate protein, we included the protein synthesis inhibitor cycloheximide [12],[56] in our studies. DEX and 5.0 µM cycloheximide treatment induced *GIK* expression at a level comparable to DEX-only treatment, implicating a direct relationship between AG and *GIK* induction in developing flowers (Figure 1E). In the time-course assay, *GIK* expression was upregulated by AG 4- and 16-fold at days 1 and 3 after AG induction, respectively (Figure 1F).

To test whether AG directly binds the *Arabidopsis* genome near *GIK*, we performed ChIP with a polyclonal antibody against AG (anti-AG) using *35S::AG-GR ag-1* inflorescences treated continuously with DEX. The primer pair hybridizing to the 3′ region of genomic *GIK* DNA containing a putative CArG box showed enrichment over a primer pair hybridizing to the coding region of *GIK* (Figure 1G). The control experiment using untreated *35S::AG-GR ag-1* inflorescences did not show obvious enrichment (Figure S2). Our data suggest that AG directly activates *GIK* by binding to the region of the *GIK* CArG box in developing flowers.

### *GIK* Is Expressed in Reproductive Organs and Is Localized to the Nucleoplasm

**Figure 2:**
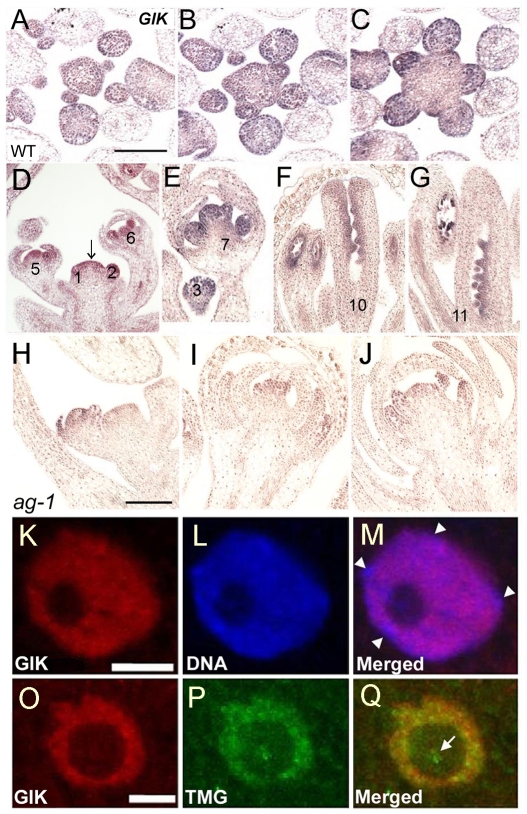
Expression pattern of *GIK* transcripts and GIK protein. (A–C) Expression of *GIK* mRNA in serial cross-sections of wild-type inflorescence meristems shown by in situ hybridization. (D–G) Expression of *GIK* mRNA in longitudinal sections of the wild-type inflorescence meristem (arrow), floral primordia, and developing reproductive organs. The numbers indicate stages of the floral buds [75]. Arrow in D indicates the inflorescence meristem. (H–J) Expression of *GIK* mRNA in *ag-1* inflorescence meristems, floral primordial, and developing flowers. (K–Q) Staining of wild-type *Arabidopsis* root cell nuclei with anti-GIK (K, O), DNA dye TOPRO-3 (L), or monoclonal anti-trimethylguanosine (TMG) (P). (M) and (Q) show merged images. Arrowheads in M indicate heterochromatin-rich chromocenters (seen as blue in the merged image). Arrow in Q indicates the nucleolus (seen as green in the merged image). Despite their largely co-localized patterns, anti-GIK staining was distinguished from that of TOPRO-3 by its lack of accumulation at the heterochromatic chromocenters (Figure 2M, arrowheads) and different from anti-TMG staining by showing no detectable expression in the nucleolus (Figure 2Q, arrow). Scale bars in A (for A–G) and H (for H–J) are 100 µm. Bars in K–Q, 5.0 µm.

To determine whether AG is responsible for *GIK* expression in reproductive development, we examined *GIK* expression in inflorescences in detail using 3′ region of *GIK* cDNA as a probe for in situ hybridization. *GIK* transcripts were detected in inflorescence meristems, floral primordia, and developing flowers (Figure 2A–G). *GIK* is expressed throughout floral primordia at stages 1 through 4 (Figure 2A–D). At stage 6 and later, *GIK* expression is confined to reproductive organ primordia (Figure 2D, E). At stages later than stage 10, *GIK* is localized in developing ovules and anther locules (Figure 2F, G). These data suggest that *GIK* expression is not fully dependent on AG (as *AG* is expressed only in the central region of flower primordia after stage 2) but that expression later than stage 6 may depend on AG during reproductive development. In *ag-1* inflorescences, although *GIK* is noticeably expressed in inflorescence meristems and floral primordia in early stages, *GIK* expression is considerably reduced in developing organs (Figure 2H–J), which is consistent with the hypothesis that *GIK* is regulated by AG in reproductive organs.

To examine GIK localization, we raised polyclonal antibodies against recombinant full-length GIK, the N-terminal and AT-hook domains. The antibody raised against full-length GIK detected a major protein band around 30 kDa in western blots (Figure S3A), in agreement with the predicted GIK protein size of 29.1 kDa (285 residues). GIK was found in roots and flowers but not significantly in leaves (Figure S3A). The level of GIK expression in roots and flowers corresponded well with our RT-PCR analysis of *GIK* transcripts in these tissues, where its expression in roots is much higher than in flowers (Figure 1C, Genevestigator: www.genevestigator.ethz.ch, AtGenExpress: www.arabidopsis.org/info/expression/ATGenExpress.jsp), suggesting that GIK might play a role in root development. To determine whether GIK is a nuclear protein, we performed immunofluorescence staining using whole-mount seedlings and confocal microscopy. Staining was specifically detected in the nucleus (Figure 2K–Q, Figure S3B) and largely colocalized with two nuclear markers: the DNA dye TOPRO-3 and the trimethylguanosine cap of small nuclear RNA (Figure 2K–Q). These results indicate that GIK is localized in the nucleoplasm. In addition, anti-GIK staining was distinguishable from both nuclear markers by a lack of GIK expression in heterochromatin chromocenters (Figure 2K–M, arrowheads, regions observed as blue color in 2M) and the nucleolus (Figure 2O–Q, arrow in 2Q).

### Overexpression and Loss of Function of *GIK* Lead to Reproductive Defects

**Figure 3:**
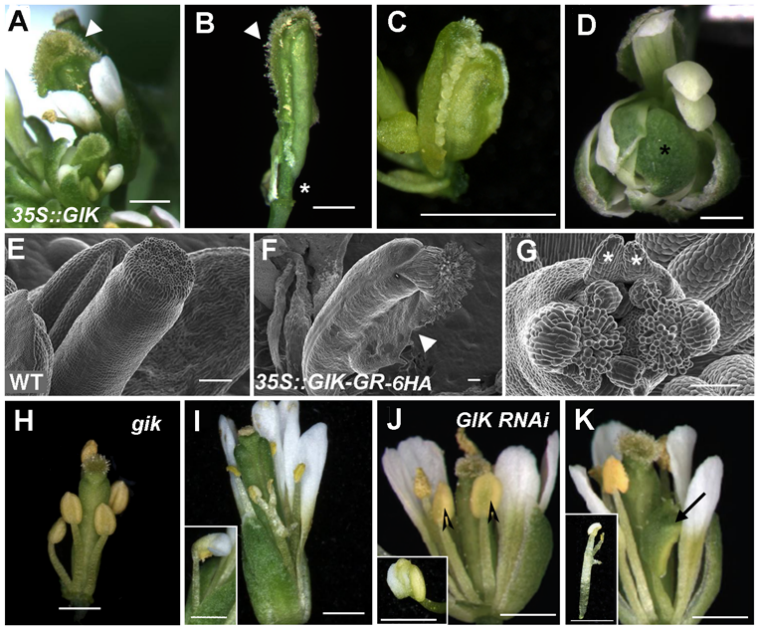
Overexpression and loss of function of *GIK* cause reproductive defects. (A–C) Flowers in *35S::GIK* overexpression plants show carpels with ectopic stigmatic tissue (marked by triangles in A, B), short valves (marked by asterisk in B), and excessive growth of carpelloid tissue at the lateral side of the pistil with exposed ovules (C). (D) Sepal-sepal fusion (asterisk) observed in *35S::GIK ag-1* flowers. (E–G) Scanning electron microscopic images of wild-type *Arabidopsis* pistil (E) and flowers from DEX-treated *35S::GIK-GR-6HA* plants show carpels with excessive stigma and ovules (arrowhead in F) and bipartite carpels with outgrowth of ovules and ectopic projections (asterisks) at the upper part of the pistil (G). (H, I) Flowers of *gik* insertion mutant *ET14389* with indehiscent anthers of stamens (H) and branched stamens (I), and defective anther differentiation showing half-petal-half-stamen morphology (inset in I). In H, sepals and petals were removed to expose inner organs. (J, K) Similar reproductive defects were observed in the flowers of *GIK RNAi* silencing lines showing delayed dehiscence (arrowheads) (J), petalloid anthers (inset in J), defective stamen formation (arrow in K), and branched stamens (inset in K). Scale bar for A–D and H–K, 1 mm. Scale bars for E–G, 100 µm.

To understand the role of *GIK* during flower development, we examined the effects of GIK overexpression. Over 20 transgenic plants from each transgenic line (*35S::GIK* and inducible *35S::GIK-GR-6HA*) were examined during flower development (Figure 3A–G). At least three T1 *35S::GIK* plants showed reduced fertility with wide-ranging defects in reproductive development such as excessive outgrowth of stigmatic tissues (Figure 3A, B), short valves (Figure 3B), and excessive proliferation of a carpelloid organ at the lateral side of a pistil with exposed ovules (Figure 3C). These phenotypes were largely recapitulated in nearly half of the T1 *35S::GIK-GR-6HA* lines after five DEX treatments (Figure 3F, G). More than 90% of flowers from the induced *35S::GIK-GR-6HA* plants showed severe reproductive defects such as excessive growth of stigmas or bipartite stigmas with outgrowth of ovules. In addition to defects in carpels, stamen development was occasionally affected, resulting in reduced male fertility (unpublished data). Similar reproductive phenotypes were observed at low frequency (3% to 4% of their flowers) in transgenic plants with a genomic copy of *GIK*, which showed 5- to 50-fold higher expression levels of *GIK* than wild-type plants (unpublished data), indicating that the *35S::GIK* and *35S::GIK-GR* constructs provide high levels of GIK activity. These results show that overexpression of GIK strongly interferes with normal reproductive development.

To examine whether GIK controls a subset of the known functions of AG, *35S::GIK* was introduced into the *ag-1* mutant plants. *35S::GIK* did not rescue the *ag-1* organ identity defects: no stamen- or carpel-like organs were observed in *35S::GIK ag-1* flowers, even though there was occasional sepal-sepal fusion (Figure 3D). This observation suggests that the function of GIK is unlike many transcription factors that control cell differentiation or specification and that instead GIK may have a unique function in modulating gene expression downstream of AG.

To further understand the role of *GIK* during flower development, a transposon insertion mutant of *GIK* (http://genetrap.cshl.edu/TrHome.html, *ET14389*) was identified. It contains an insertion in the middle of the coding region, 450 bp from the start codon. Homozygous plants were verified by PCR-based genotyping and *GIK* expression analysis (Figure S4A, B). Most of the flowers from *gik* homozygous mutants appeared normal without any gametophytic defects (unpublished data), but a small number of flowers (22 of 800) showed various degrees of defects in stamen and carpel development (Figure 3H, I, Figure S4C–E). Stamen development was impaired, which resulted in delayed dehiscence or indehiscence of anthers (Figure 3H). In some cases, the filaments of the stamens were branched and had ectopic anther formation, and anthers were partially transformed into petal-like structures (Figure 3I, Figure S4C, D). None of the defects were observed in wild-type plants grown under the same conditions. To examine whether the mutant phenotypes were caused by loss of GIK activity, we generated RNA interference (RNAi) silencing lines using the 3′ end of the *GIK* coding region (Figure 3J, K). In 5 of 29 independent T1 RNAi plants, we observed similar defects of immature anthers and branching of stamen filaments at a similarly low frequency in T1 and T2 generations. We confirmed that the *GIK* transcripts were significantly reduced in flowers of *GIK RNAi* plants (unpublished data). To examine whether *GIK* has redundant functions with other *GIK*-like genes [32],[33], we produced an RNAi silencing construct for the highly similar gene *At4g17800* (67% amino acid identity) and created the transgenic plants on the *gik* mutant background. However, we did not observe any obvious enhanced effects in the transgenic plants (unpublished data). The GIK loss-of-function defects, albeit not at a high frequency, suggest some level of participation by GIK in reproductive development as a component of a fine-tuning mechanism.

### GIK Negatively Regulates *ETTIN* Expression

**Figure 4:**
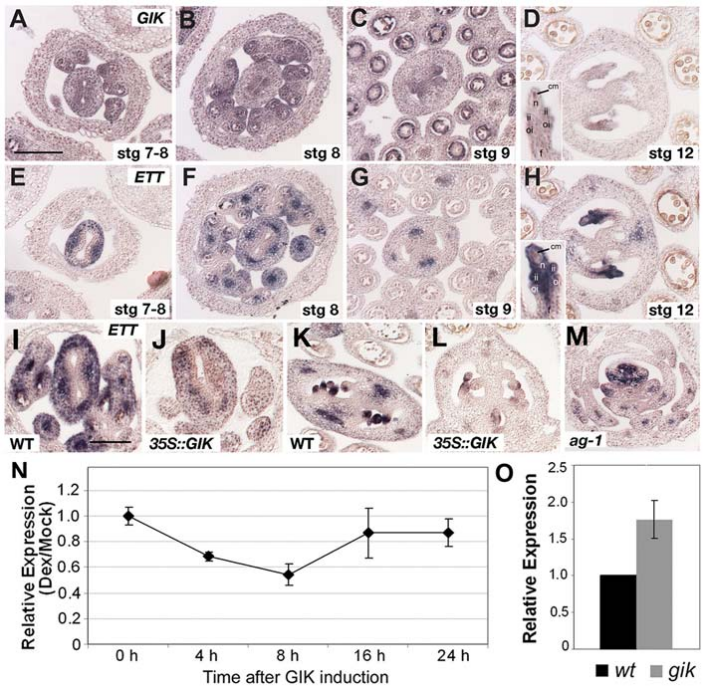
*GIK* negatively regulates *ETT*. (A–H) *GIK* (A–D) and *ETT* (E–H) exhibit complementary expression patterns in reproductive organs at stages 7–8 (A, E), 8 (B, F), 9 (C, G), and 12 (D, H) as shown by in situ hybridization. At stages 7, 8, and 9, *GIK* is expressed at the adaxial side of the developing carpels and locules of developing stamens (A–C). In contrast, *ETT* is expressed at the abaxial sides of the carpels and in the vasculature of the stamens (E–G). At stage 12, *GIK* expression was mainly observed in the funiculus (f), outer integument (oi), and chalazal megaspore (cm) of ovules (D, inset), whereas *ETT* expression was in inner integuments (ii) and the nucellus (n) of the ovules (H, inset). (I–L) Comparison of *ETT* expression in the reproductive organs of wild-type (I and K) and *35S::GIK* (J and L) plants by in situ hybridization on a single slide. (M) *ETT* expression in an *ag-1* mutant flower. Scale bars in A (for A–H) and I (for I–M) are 100 µm. (N) Time-course of *ETT* expression after GIK activation, as measured by real-time PCR. Inflorescences from *35S::GIK-GR-6HA* plants were harvested at 0, 4, 8, 16, and 24 h after mock treatment or a single DEX treatment. *ETT* expression was normalized to *TUB* RNA levels. Relative expression in DEX-treated samples was calibrated with mock-treated samples. (O) Expression analysis of *ETT* in the *gik* mutant using real-time PCR with RNA extracted from the inflorescences of wild-type and *gik* mutant *ET1438*9 plants. Expression was normalized to *TUB* expression. Relative expression level in the wild-type was set to 1.0. Standard deviation was obtained from three independent biological samples in N and O.

Because GIK overexpression phenotypes of outgrowth of stigmatic tissues, short valves, and bipartite stigmas with ectopic ovule formation (Figure 3A–C, F, and G) closely resemble loss-of-function phenotypes of the previously identified *ettin* (*ett*, meaning “giant”) mutants [19]–[21],[57], we examined whether there is a functional link between *GIK* and *ETT*. *ETT* encodes a member of an auxin response factor family of DNA binding proteins, and loss of ETT activity results in severe reproductive defects [20],[57]. First, we crossed *35S::GIK-GR-6HA* plants with the weak *ett-3* mutant. Overexpression of *GIK* in the heterozygous and homozygous backgrounds of the weak *ett-3* allele showed strong *ett* mutant phenotypes (Figure S5), suggesting an epistatic interaction of *GIK* overexpression with *ETT*. Next, we compared the expression patterns of *GIK* and *ETT* in wild-type reproductive organs in detail using *in situ* hybridization analysis (Figure 4A–H). At floral stages 7–12, *GIK* and *ETT* exhibited complementary expression patterns in the developing reproductive organs (*GIK*, Figure 4A–D; *ETT*, Figure 4E–H). *GIK* is predominantly expressed in the adaxial part of the developing carpels and locules of stamens (Figure 4A–C). On the other hand, *ETT* is expressed in the abaxial part of the developing carpels and in the vasculature of the stamens (Figure 4E–G). In the developing ovules, complementary expression of *GIK* and *ETT* was also apparent (Figure 4D, H): *GIK* was mainly expressed in funiculi, outer integuments, and the chalazal megaspores (Figure 4D), whereas *ETT* expression was restricted to inner integuments and the nucellus (Figure 4H).

Next, we compared *ETT* expression in wild-type and *35S::GIK* plants (Figure 4I–L). *ETT* signals in the *35S::GIK* flowers were lower than in the wild-type flower sections when stained on the same slide (compare Figure 4I and J, 4K and L). Because the overexpression phenotype of *GIK* can be interpreted as the repression of *GIK* by ETT, we tested this possibility by examining *GIK* expression in *ett* mutant flowers. However, *GIK* expression was not upregulated in *ett* mutant flowers (Figure S6). Taken together, these results suggest that GIK can negatively regulate *ETT*, but not vice versa.

Next, we examined *ETT* expression in the flowers of *ag-1* mutants and inducible AG lines. *ETT* expression was only observed in the abaxial sides of early organ primordia and was not maintained in maturing organs in an *ag* mutant background (Figure 4M). This indicates that late *ETT* expression, which can be modulated by GIK, requires AG activity. In *ag-1 35S::AG-GR* inflorescences, *ETT* expression was reduced to 80% of the initial level at 1 d after AG induction and then upregulated from day 2 onwards (Figure S7). These results suggest that *ETT* expression is positively regulated by AG, but at the same time, negatively modulated by GIK.

To examine the regulatory effects of GIK on *ETT* in detail, time-course analysis of *ETT* expression was performed with *35S::GIK-GR-6HA* transgenic plants with inducible GIK activity using real-time PCR. *ETT* expression was downregulated 4 h after a single DEX treatment that induces GIK activity, reached its lowest level at 8 h, and then returned to pretreatment levels (Figure 4N), suggesting that induced GIK activity rapidly repressed *ETT* expression and that the *ETT* repression requires continuous GIK expression. Furthermore, we quantitatively measured *ETT* expression levels in *gik* mutant flowers. *ETT* was upregulated about 1.8 times in *gik* mutant flowers as compared to wild-type (Figure 4O). These results suggest that GIK functions as an upstream negative modulator of *ETT* at certain floral stages.

### GIK Is a Bona Fide Matrix Protein and Binds *ETT* Putative MARs In Vitro and In Vivo

**Figure 5:**
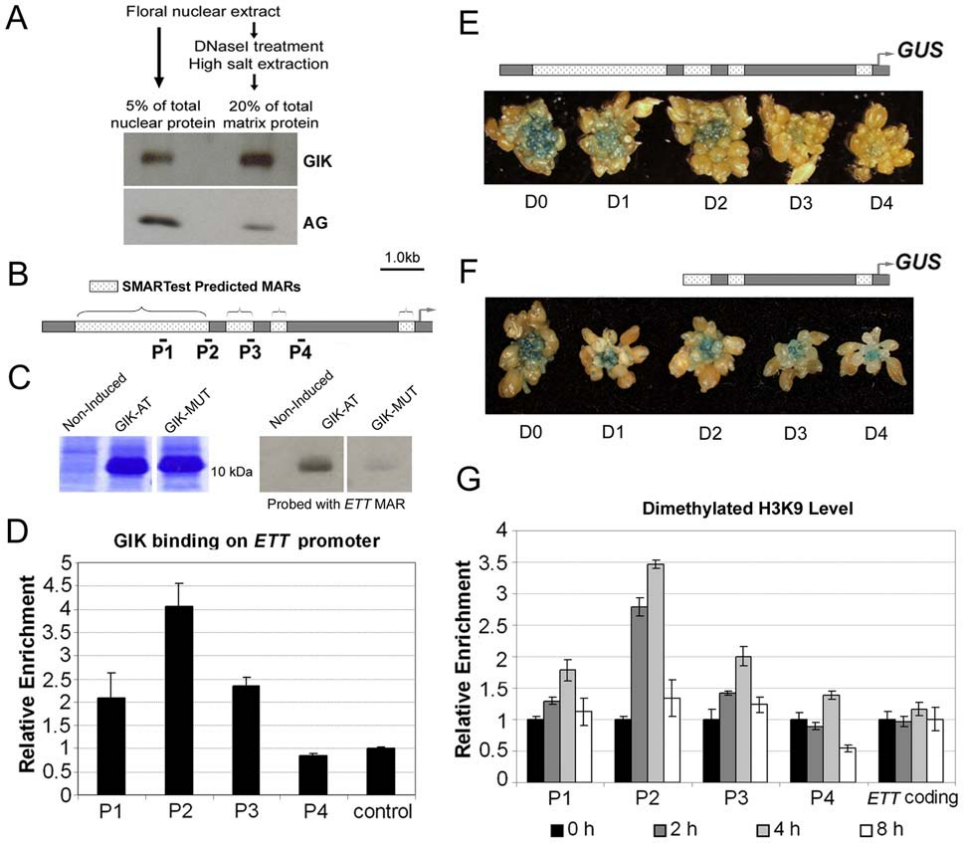
GIK binds to putative MARs of *ETT* genomic DNA to modulate its expression. (A) GIK is localized to the nuclear matrix. Nuclear matrix was isolated from the inflorescences of *35S::GIK-GR-6HA* plants treated with DEX and harvested 4 h thereafter. Total nuclear and matrix proteins were subjected to western blot analysis. The membrane was first probed with anti-HA to detect GIK and then re-probed with anti-AG. (B) Schematic representation of SMARTest-predicted MARs in the *ETT* upstream genomic region. Arrow indicates the transcription start site. P1, P2, P3, and P4 are primer pairs used to detect different regions of the *ETT* genomic DNA used in the ChIP assay. (C) In vitro MAR binding assay of GIK. Left panels, Coomassie Blue staining of a gel loaded with non-induced *E. coli* containing the wild-type GIK AT-hook motif construct (noninduced), with an IPTG-induced culture containing the construct for the wild-type GIK AT-hook motif (GIK-AT), and with IPTG-induced culture containing a mutated construct in the conserved residues of the GIK AT-hook motif, changing Arg-Gly-**Arg**-Pro to Arg-Gly-**Lys**-Pro (GIK-MUT). Right panels, the corresponding south-western results of the MAR binding assay probed with an *ETT* MAR probe. (D) GIK binds to the MARs of the *ETT* promoter in vivo. Inflorescences from *35S::GIK-GR-6HA* plants treated with DEX were harvested 4 h after DEX induction for ChIP experiments. Anti-HA was used for immunoprecipitation. Relative enrichment was obtained from the ratio of enrichment achieved by anti-HA to that of control IgG. Enrichment of a sequence amplified from the *TUB* locus was used as a basal control and set to 1.0. P1, P2, P3, and P4 are primer pairs used to detect different regions of the *ETT* genomic DNA (as illustrated in B). (E, F) Time-course promoter analysis of the *ETT* gene after GIK induction. *35S::GIK-GR-6HA* transgenic plants were crossed with plants transgenic for promoter constructs of wild-type *pETT::GUS* (E) and *pETTΔMAR::GUS* with a deletion of distal MARs (F). The inflorescences were treated continuously with DEX every 2 d and harvested for GUS staining at 0, 1, 2, 3, and 4 d after the initial DEX treatment. Upper panels, schematic representations of the *ETT* upstream genomic region fused with a *GUS* reporter gene. Lower panels, GUS-stained inflorescences at 0, 1, 2, 3, and 4 d after the initial GIK induction. (G) Time-course analysis of dimethylated-H3K9 level associated with the *ETT* genomic DNA in *35S::GIK-GR-6HA* inflorescences at 0, 2, 4, and 8 h after a single GIK induction. ChIP was performed using anti-dimethylated H3K9 (Upstate). Primer pairs P1, P2, P3, and P4 are shown in Figure 5B. Relative enrichment was obtained from the ratio of bound/input achieved in the respective time points to that at 0 h. The bound/input ratio was first normalized with the bound/input ratio of a basal control, *PFK*, the transcription of which is not affected by GIK. The enrichment at 0 h was set as 1.0. Standard deviation was obtained from PCR triplicates in D and G.

GIK contains an AT-hook DNA binding motif, which binds to the MAR of DNA sequences [36],[38]. To examine how GIK controls *ETT* expression, we first examined whether GIK is a bona fide nuclear matrix-bound protein. Because the endogenous expression level of GIK in inflorescences is low (Figure 1C, Figure S3A), we used inflorescences from *35S::GIK-GR-6HA* plants. We isolated the nuclei from the inflorescences of DEX-treated *35S::GIK-GR-6HA* plants (the inflorescences were harvested 4 h after DEX treatment) and then purified the matrix fraction by DNaseI treatment and extensive washing with high-salt buffer, which removes basic proteins and histones [58],[59]. The total nuclear protein and the matrix fraction were probed with anti-HA that recognizes GIK-GR-6HA protein (Figure 5A). A strong GIK signal was observed in the matrix fraction of the nuclei. In comparison, AG (as a control) was mostly washed away during the extraction processes, and only a faint signal was detected in the matrix fraction on the same membrane (Figure 5A). This suggests that GIK is associated with the nuclear matrix.

Next, to examine whether there are putative binding sites for GIK in the *ETT* promoter, we identified MARs in the upstream genomic region of *ETT* using SMARTest Software (Figure 5B) [60]. To test whether GIK can bind the putative MARs in the *ETT* promoter region, we expressed a truncated GIK with an intact AT-hook motif in *E. coli* and checked for its binding to an *ETT* putative MAR probe. We detected binding of the *ETT* probe to the GIK AT-hook domain (Figure 5C, GIK-AT). The binding activity was reduced when one of the conserved binding regions, Arg-Gly-Arg-Pro (Figure 1B) [36] of the AT-hook domain, was mutated to Arg-Gly-Lys-Pro (Figure 5C, GIK-MUT), suggesting that the wild-type AT-hook motif binds to the predicted MAR in the *ETT* promoter in vitro.

To examine whether GIK binds to the putative MARs of the *ETT* promoter in vivo, we performed a ChIP assay using inflorescences from *35S::GIK-GR-6HA* plants. The plants were treated with DEX, and the inflorescences were harvested 4 h later. Nuclear proteins were solubilized by sonication and immunoprecipitated with anti-HA. The putative MARs of the distal *ETT* promoter, especially the region represented by primer set P2, showed clear enrichment (Figure 5D). In contrast, neither the region that is close to one of the predicted MARs represented by primer set P4 nor the control showed enrichment (Figure 5D). To examine whether endogenous GIK binds the putative MARs of the *ETT* promoter in a non-transgenic context, we repeated the ChIP experiment using wild-type inflorescences and the polyclonal anti-GIK. The result, albeit with some differences in the fold enrichment, indicated that GIK binds to the putative MARs of the distal *ETT* promoter in vivo (Figure S8).

### The *ETT* MAR Is Necessary for GIK-Mediated Repression

To evaluate whether the binding of GIK to the putative MARs of the *ETT* promoter is necessary for *ETT* regulation, we performed *ETT* promoter-reporter analysis (Figure 5E, F, Figure S9). We generated transgenic reporter lines in which the major MAR (represented by primer sets P1 and P2), located at distal part of the *ETT* upstream genomic region, was deleted (*pETTΔMAR::GUS*) (Figure 5F). As a control, the upstream genomic region of *ETT* inclusive of all MARs (*pETT::GUS*) was fused with a *GUS* reporter gene and the inflorescences were stained (Figure 5E). Expression of GUS in T1 *pETTΔMAR::GUS* transgenic lines was comparable or slightly weaker compared with that of *pETT::GUS* lines (Table S3). This result suggests that *ETT* expression is normal even after the deletion of these 5′ distal regions, and that the deleted regions may not contain regulatory elements or may contain both positive and negative regulatory elements for transcription. These reporter lines were crossed with the *35S::GIK-GR-6HA* plants to test their responsiveness to ectopic GIK activation. There was a gradual reduction of GUS activity in response to continuous DEX treatment in plants transgenic for the construct with a full-length *ETT* promoter (*pETT::GUS*) in a time-dependent manner (Figure 5E). At day 3 and later, GUS staining was barely detectable. In contrast, the *pETTΔMAR::GUS* reporter line was less responsive to GIK (Figure 5F). To exclude the possibility that the no responsiveness is due to positional effects of an insertion site, we repeated the experiments using an independent line and confirmed that *pETTΔMAR::GUS* reporter line does not respond to GIK activity (Figure S9A). To quantify this MAR-dependent repression of GUS activity by GIK, we carried out time-course *GUS* reporter gene expression analysis using quantitative real-time PCR (Figure S9B, C). In agreement with the reduction in GUS staining, *GUS* expression in the *pETT::GUS* line was significantly downregulated at days 3 and 4 after GIK induction, respectively (Figure S9B). In contrast, in the *pETTΔMAR::GUS* reporter line, there was no significant reduction of *GUS* expression at day 4, and in fact a slight increase was seen at day3 (∼1.3-fold) after GIK induction (Figure S9C). These results suggest that repression of *ETT* by GIK requires the sequence containing the distal putative MARs of the *ETT* promoter.

### GIK-Mediated *ETT* Repression Is Associated with Dynamic Changes in Dimethylated Histone H3 at Lys9

To examine whether repression of *ETT* is associated with any known epigenetic histone modifications, we performed a ChIP assay using antibodies against modified histones in wild-type and *35S::GIK* backgrounds (for details, see Materials and Methods). One of the repressive marks, dimethylated Lys 9 of histone H3 [61], was found to be specifically enriched in the *35S::GIK* background in the *ETT* upstream region (Figure S10). To gain further insight into the change in H3K9 dimethylation, we performed a time-course ChIP analysis using inflorescences of *35S::GIK-GR-6HA* plants treated one time with 10 µM DEX. We observed a rapid increase in H3K9 dimethylation at the distal portion of the putative MAR within 2 h of GIK induction, especially in the region represented by primer set P2 (Figure 5G). At 4 h post-induction, the increase in H3K9 dimethylation reached a maximum, with a 3- to 4-fold increase in the dimethylation level in the *ETT* upstream region (Figure 5G). This change in dimethylated H3K9 was relatively rapid and dynamic: at the 8 h time point, the level was comparable to that at time 0. *ETT* transcript levels were reduced to their lowest levels at the 8 h time point after *GIK* induction (Figure 4N). This result suggests that the GIK-mediated *ETT* change requires continuous GIK activity and that the repression is closely associated with a dynamic change in the extent of H3K9 dimethylation in the *ETT* upstream region.

### GIK Regulates a Set of Reproductive Genes

**Figure 6:**
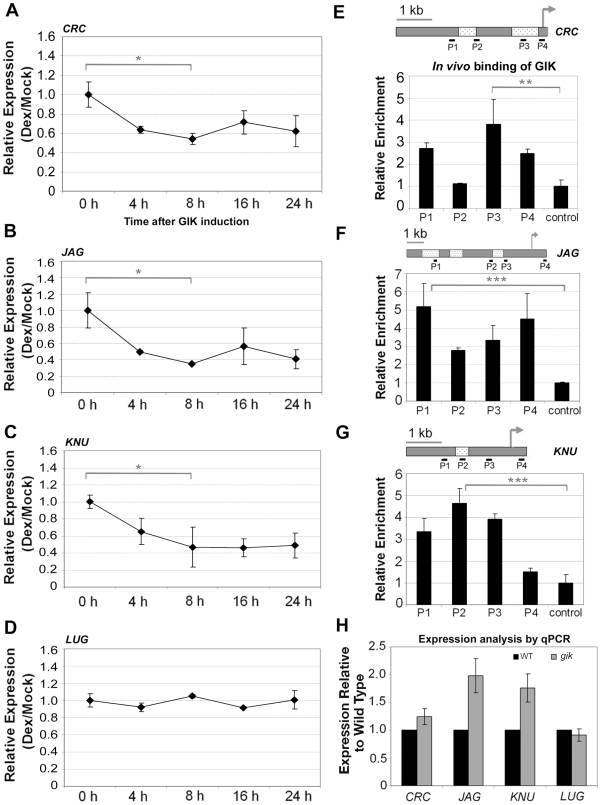
*GIK* regulates multiple reproductive regulators. (A–D) Time-course expression analysis of *CRC* (A), *JAG* (B), *KNU* (C), and *LUG* (D) transcripts upon GIK activation. Inflorescences from *35S::GIK-GR-6HA* plants were harvested at 0, 4, 8, 16, and 24 h after a single DEX treatment for quantitative real-time PCR. Target gene expression was normalized to *TUB*. Relative expression in DEX-treated samples was calibrated with mock-treated samples. Standard deviation was obtained from three independent biological samples. The differences between 0 h and 8 h were statistically analyzed using paired student's *t*-test. *p<0.05 in (A), (B), and (C). *p*>0.1 in (D). (E–G) GIK binds the upstream MAR regions of *CRC* (E), *JAG* (F), and *KNU* (G) genomic DNA in vivo. Schematic representations of genomic regions of these genes are shown with demarcated SMARTest-predicted MAR regions. Primer sets used for quantitative PCR are shown below each graph. Arrows indicate transcription start sites. Relative enrichment was obtained from the ratio of enrichment achieved by anti-HA to that of control IgG. The enrichment value obtained from a sequence amplified from the *TUB* locus is shown as a control and set to 1.0. Standard deviation was obtained from PCR triplicates. The differences between the control and the primer pairs showing the highest enrichment were statistically analyzed using student's *t*-test. **p<0.1 in (E), ***p<0.05 in (F) and (G). (H) Expression analysis of *CRC*, *JAG*, *KNU*, and *LUG* in the *gik* mutant using real-time PCR as in Figure 4O.

To account for the pleiotropic phenotypes conferred by overexpression and loss of function of *GIK* (Figure 3), we examined a panel of reported *Arabidopsis* genes involved in reproductive development for their expression responses to GIK using real-time PCR (Figure 6A–D and Table S4). Many genes including *LUG*, which is a putative repressor of *AG* and whose loss of function leads to bipartite stigmas [22]–[24], showed no clear changes in expression upon GIK activation in the time-course experiments using *35S::GIK-GR-6HA* inflorescences (Figure 6D, Table S4). However, expression of *CRC*, *JAG*, and *KNU* decreased significantly after GIK induction (Figure 6A–C).

To determine whether GIK directly regulates *CRC*, *JAG*, or *KNU*, we first examined transcriptional repression by including the protein synthesis inhibitor cycloheximide (Figure S11). DEX with cycloheximide treatment repressed *ETT*, *CRC*, *JAG*, and *KNU* expression in 2 h at a level comparable to DEX-only treatment, indicating that transcriptional repression by GIK does not require de novo protein synthesis (Figure S11). In the upstream region of each of these genes, one to three predicted MARs were identified using SMARTest (Figure 6E–G). The prediction made by the SMARTest program could contain false-positive and false-negative results (Figure 5D) [60]. To validate the SMARTest prediction, we performed ChIP experiments and showed apparent enrichment using primer sets that detect some of the putative MAR regions of these target genes (Figure 6E–G). In the *CRC* promoter, there are two predicted MARs. Both the distal and proximal putative MARs showed a clear enhanced binding compared with control, whereas primer set P2, which amplifies the 3′ region of the distal putative MAR, showed no clear enrichment (Figure 6E). In the *JAG* promoter, the most distal of the three putative MARs showed the strongest enrichment (Figure 6F). The 5′ transcribed region of *JAG* showed an unexpectedly high enrichment, which may indicate that an unpredicted MAR site is located in the transcribed region of *JAG*. In the *KNU* promoter, there was only one predicted MAR, and the enrichment index showed a bell-shaped distribution centered on the binding site (Figure 6G). These results suggest that ectopically expressed GIK binds directly to the putative MARs of these target genes and represses their transcription.

**Figure 7:**
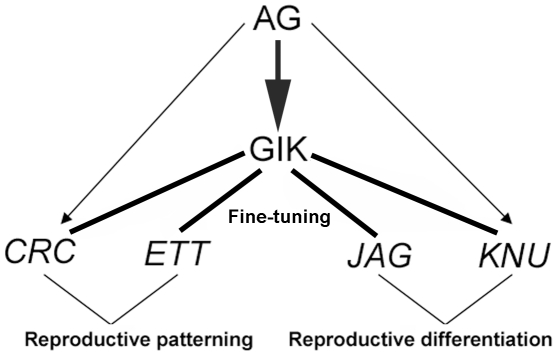
Summary diagram of GIK regulation and function. The MAR binding protein GIK is directly regulated by the floral homeotic protein AG during reproductive development. GIK modulates and refines the expression of *ETT* and *CRC* to control reproductive patterning and *JAG* and *KNU* for reproductive differentiation. GIK functions as a multifunctional determinant to coordinate gene expression during reproductive development.

To determine whether endogenous GIK is involved in the regulation of *CRC*, *JAG*, or *KNU*, we examined the expression of these genes in the *gik* mutant background. Real-time PCR using flowers from *gik* homozygous plants showed that *JAG* and *KNU* were relatively highly expressed in the *gik* mutant (Figure 6H). Expression of *CRC* was slightly increased, but expression of *LUG* was not changed in the *gik* mutant. These results suggest that *GIK* is involved in a mode of regulation that ensures proper levels of expression of multiple genes during reproductive development (Figure 7).

## Discussion

The floral homeotic protein AG is a key determinant of reproductive organ development. AG is thought to control the spatiotemporal expression of over 1,000 genes responsible for stamen and carpel development [9]. We showed that AG regulates the expression of *GIK*, which codes for a MAR binding protein with an AT-hook DNA binding motif. We further demonstrated that the expression of various key transcription factors in flower development, including *ETT/ARF3*, is modulated directly by GIK by binding to the putative MARs. We also showed that GIK modulation of target genes is closely associated with epigenetic modifications. Our data strongly suggest that GIK has multiple inputs into transcriptional control of reproductive development downstream of AG (Figure 7).

### *GIK* Expression Balances Patterning and Organogenesis

Although organ patterning and organogenesis are generally thought to occur independently, evidence has emerged that there is cross-talk between these processes. The homeotic protein AG controls stamen identity partly by activating *SPL/NZZ*, a gene necessary for specification of male gametophytes [12]. During late stamen development, AG directly controls *DAD1* to induce jasmonic acid biosynthesis for stamen maturation [18]. Here we show that another target of AG, *GIK*, modulates the expression of the auxin response factor *ETT* through epigenetic modification of the *ETT* promoter. *ETT* controls patterning in both the abaxial-adaxial and apical-basal axes of reproductive organs [19]–[21],[57]. GIK also influences the expression of other key regulators during reproductive development, such as *CRC*, another abaxial-adaxial polarity-controlling *YABBY* family gene [25],[26]; *JAG*, which is involved in proliferation and differentiation of carpels [28]; and *KNU*, which is involved in floral meristem determinacy and gametophyte differentiation [30]. Thus, we propose that organ patterning that is mediated by *ETT* (and possibly *CRC*) and reproductive differentiation that is regulated by *KNU* and *JAG* are under partial control of AG, and that GIK acts as a molecular organizer to orchestrate expression of these key regulators for floral reproductive patterning and differentiation (Figure 7).

Ectopic *GIK* expression in the *ag-1* mutant background had minor effects on organ identity and patterning. This does not, however, imply that *GIK* has no clear function as an AG target. Rather, our data suggest that GIK may modulate and refine spatial and temporal expression of multiple genes downstream of AG. The direct GIK targets, *ETT*, *CRC*, *JAG*, and *KNU*, are predominantly expressed in reproductive organs, and their expression depends on AG activity to varying degrees. *CRC* and *KNU* are directly regulated by AG [13],[31]. *ETT* locus is directly bound by SEPALLATA3, a binding partner of AG [14]. Thus, the effects of ectopic GIK expression were only observed in the wild-type context in which genes downstream of AG are activated. Based on this observation, we conclude that the general role of GIK is to fine-tune the expression of key regulators necessary for patterning and differentiation during reproductive development (Figure 7).

We observed a relatively low penetrance of *GIK* loss-of-function phenotypes, despite robust phenotypes caused by *GIK* overexpression. In addition to a possible redundancy, this observation may suggest that *GIK* does not act as a steadfast controller of gene expression but rather that it fine-tunes the expression of multiple genes through chromatin formation. Furthermore, GIK is expressed in tissues other than flowers, with especially robust expression in roots. Therefore, GIK may have an AG-independent and root-specific function during root growth and development. In agreement with this observation, overexpression of GIK caused root growth inhibition (Figure S12), even though loss of GIK function did not show clear morphological defects in roots (unpublished data).

### Regulation of *ETT* by GIK

We showed that *ETT* is a major target gene for repression by GIK during reproductive development based on the results of a series of genetic and molecular experiments: (1) GIK overexpression mimics the phenotypes of *ett* mutants, (2) *GIK* and *ETT* show complementary expression patterns during late reproductive development, (3) *ETT* expression is increased in *gik* mutants, (4) GIK binds to *ETT* putative MARs in vivo, and (5) the putative *ETT* MARs are important in GIK-regulated *ETT* expression. GIK-mediated repression of *ETT* occurred relatively rapidly after GIK induction in floral tissues, and the stable repression of *ETT* required continuous GIK activity. We also showed that *ETT* silencing was associated with repressive histone dimethylation of H3K9 in the *ETT* promoter, especially at the distal putative MAR. It remains unclear whether GIK is directly involved in this histone modification. Because GIK lacks known domains typically found in chromatin-modifying enzymes, GIK may introduce structural changes to the genomic region through MAR binding and may thereby facilitate the binding of chromatin-modifying enzymes to carry out histone modifications. It is also possible that GIK serves as a center for organizing chromatin remodeling complexes in the nuclear matrix to regulate target gene expression. Alternatively, MAR binding by GIK may inhibit the binding of the transcriptional machinery to the proximal promoter, leading to gene silencing associated with dimethylated H3K9. However, our time-course analysis showed that the dynamic changes in H3K9 dimethylation levels appeared to precede negative regulation of *ETT* transcription, which does not support the later hypothesis. Dimethylation of H3K9 increased rapidly during the 2 h after GIK induction. A further increase in dimethylation at the 4 h time point corresponded with the steepest downregulation of *ETT* transcription at the 8 h time point. Conversely, a dynamic reduction of H3K9 dimethylation to a level lower than that seen prior to induction at the 8 h time point was followed by a steady recovery of *ETT* transcription at the later time points of 16 h and 24 h. Nevertheless, how this dynamic methylation pattern is achieved remains unknown. The mammalian AT-hook protein SATB1 has been shown to mediate gene repression by directly recruiting histone deacetylases [62]. Further studies of proteins that interact with GIK may provide a more detailed account of the mechanism of GIK-mediated repression.

*ETT* has recently been shown to be regulated by trans-acting short interfering RNAs (siRNAs) [63]–[65]. Interestingly, *ETT* expression may be refined by two different molecules, GIK and siRNA, to establish strict spatiotemporal expression boundaries. These events may also partially explain the modest effects of the *GIK* loss-of-function mutant and of deletion of putative MAR regions in the *ETT*-promoter reporter construct in the wild-type context. However, it remains to be determined whether GIK and siRNA have separate or overlapping roles in the control of *ETT* in reproductive development.

### Evolutionary Convergence on AT-Hook Motif MAR-Binding Proteins

In mammals, MAR-binding proteins have been implicated in the control of expression of multiple genes. SATB1 in mice contains an AT-hook DNA binding motif and acts as a “gene organizer” to regulate temporal and spatial expression of multiple genes during thymocyte maturation and breast tumor growth and metastasis [40],[44]. Another SATB1-related MAR-binding protein, SATB2, represses the expression of several *Hox* genes during skeletal development and osteoblast differentiation [43]. In agreement with these studies, we show that GIK exhibits similar properties in its regulation of target genes. First, these proteins share the role of a matrix binding protein with an AT-hook DNA binding motif and regulate expression of multiple genes. Second, they are important regulators of various developmental processes: SATB1 in T-cell development, SATB2 in craniofacial patterning and osteoblast differentiation, and GIK in floral reproductive development. Third, most of these proteins execute their effects by modifying chromatin (SATB1 recruits histone deacetylase, whereas negative regulation by GIK is associated with H3K9 methylation). Thus, convergent evolution may have permitted proteins with the same motif to be used for transcriptional coordination in the two kingdoms. Plants and animals are considered to have independently evolved their multicellular developmental processes, but organ or segment identity control in plants and animals starts with transcription factors: *HOX* genes in animals and *MADS* genes in plants [66]. Proteins with AT-hook motifs are predominantly present in eukaryotes. The motif is found in some families of HMG proteins that bind to the minor groove of DNA, and the proteins may serve as an anchor for chromatin modifying proteins or may change chromatin architecture [37],[38],[62]. Such properties may explain why AT-hook proteins have been used in the evolution of both plant and animal development. In mice, SATB2 controls the expression of the homeotic protein Hoxa2 [43]. In contrast, the homeotic protein AG controls the expression of GIK in *Arabidopsis*. Thus it is possible that AT-hook motif proteins have been independently incorporated into multicellular developmental processes in animals and plants, but with similar functions of orchestration and fine-tuning of tissue-specific expression of multiple genes.

## Materials and Methods

### Plant Materials and Chemical Treatments

All plants used in this study are on the Landsberg *erecta* background and were grown at 22°C under continuous light. DEX treatment was done by submerging inflorescences in a solution containing 10 µM DEX together and 0.015% Silwet L-77 for ∼1 min. Transgenic plants were generated by *Agrobacterium*-mediated infiltration [67]. Plant photographs were taken using a Nikon SMZ 1500 stereoscopic microscope attached to a digital camera (SIGHT DS-U1). Scanning electron microscope images were taken using a JEOL JSM-6360LV scanning electron microscope.

### Generation of *GIK* Loss of Function, Complementation, RNAi, and Overexpression Lines

To generate the *35S::GIK* and *35S::GIK-GR-6HA* constructs, *GIK* cDNA was cloned into a *pMAT137* vector and a composite *pGreen* vector containing a rat GR hormone binding domain and a 6×HA tag, respectively [12],[68]. Transgenic plants were selected with kanamycin (for the *pMAT137* construct) and BASTA (for the *pGreen* construct) for two generations to obtain homozygous lines. *35S::GIK-GR-6HA* plants were treated with DEX five times at 1 d intervals for phenotypic observation. More than 90% of DEX-treated flowers showed reproductive defects. A *GIK* insertion line was obtained from the TRAPPER collection (http://genetrap.cshl.edu/TrHome.html) (NASC stock number, *ET14389*). The enhancer trap was inserted into the middle of the coding region, 450 bp downstream from the start codon. Homozygous lines were verified by PCR-based genotyping. In total, ∼1% of *gik* mutant flowers showed reproductive defects. For the rescuing experiment of the *gik* mutant, a genomic copy of *GIK*, containing 4,660 bp of the 5′ upstream region, 858 bp of the *GIK* coding region, and 1,767 bp of the 3′ region, was cloned into the *pDONR221* vector (Invitrogen) and later into the *pBGW* binary vector using gateway cloning [69] for plant transformation. Unexpectedly, we obtained lines showing 5–50-fold higher expression levels of *GIK*, thus showing the ectopic expression phenotypes. To generate the *35S::GIK-RNAi* construct, a C-terminal fragment of the *GIK* coding region (*GIK-Cter*, 410–808 bp) was amplified using UltraPfu-High-Fidelity DNA polymerase (Stratagene) to produce *BamHI-GIK-Cter-ClaI* and *XhoI-GIK-Cter-KpnI* fragments. These fragments were cloned into the *pKANNIBAL* vector [70]. *pKANNIBAL-GIK-RNAi* was cut by *NotI* to produce a *35S::GIK-RNAi* fragment, which was then cloned into the *pMLBART* binary vector [71]. *GIK-RNAi* transgenic plants were selected using BASTA. A few percentages of the examined flowers showed reproductive defects in the T1 and T2 generations. In the T3 generation, the lower ratio of the *GIK-RNAi* flowers showed reproductive defects. To generate the *35S::GIK2-RNAi* construct, an N-terminal fragment of the *GIK2* (*AT4g17800*; 39–260 bp) coding region was amplified using UltraPfu-High-Fidelity DNA polymerase to produce *BamHI-GIK2-Nter-ClaI* and *XhoI-GIK2-Nter-KpnI* fragments. These fragments were cloned into the *pKANNIBAL* vector and later into the *pMLBART* binary vector as described in the cloning process for *35S::GIK-RNAi*. A T1 *35S::GIK2-RNAi* plant was crossed to *gik* and the *GIK2 RNAi gik* plants were obtained and confirmed following BASTA selection and PCR genotyping.

### Antigen Purification and Polyclonal Antibody Production

Full-length *GIK* cDNA and cDNAs of the conserved N-terminal and AT-hook domains were cloned into the *pQE30* vector (QIAGEN) to produce 6×His-GIK proteins. Recombinant protein was induced using 1 mM IPTG and purified on a nickel column (QIAGEN) under denaturing conditions. Protein was then partially refolded through buffer exchange and concentrated using a Centriprep Centrifugal Filter with an Ultracel YM-10 membrane (Millipore). Purified 6×His-GIK recombinant protein was injected intramuscularly into guinea pigs with Freund's adjuvant. Blood was withdrawn after the fourth and sixth immunizations. Whole blood was processed to obtain polyclonal anti-GIK serum.

### Western Blot Analysis

Approximate 0.035 g each of *Arabidopsis* roots, flowers, and leaves was ground in liquid nitrogen and re-dissolved in 80 µL SDS sample loading buffer (0.125 M Tris-HCl, pH 6.8, 4% SDS, 10% β-mercaptoethanol, 20% sucrose, 0.02% bromophenol blue). The samples were boiled for 10 min, and 25 µL of each sample was loaded onto a 12% SDS polyacrylamide gel for electrophoresis. Proteins were transferred onto a PVDF nylon membrane (Bio-Rad) and blocked with skim milk. The membrane was then incubated overnight with polyclonal anti-GIK at 4°C, washed with 20 mM Tris-HCl, pH 7.5, 137 mM NaCl, and 0.1% [v/v] Tween 20 and further incubated with secondary anti-guinea pig coupled to horseradish peroxidase. Signal was detected using SuperSignal West Dura extended duration substrate (Pierce). A replicate membrane was stained with Coomassie Blue to show protein loading.

### Immunofluorescence Staining

*Arabidopsis* seedlings were rinsed with 1× phosphate buffered saline (PBS) and fixed with 4% paraformaldehyde in PBS for 1 h. Seedlings were washed three times with PBS and incubated with 4% Driselase (Sigma) at 37°C for 30 min. After washing, seedlings were further incubated with PBS containing 10% dimethyl sulfoxide and 3% [v/v] NP-40 for 1 h at room temperature. Seedlings were washed three times with PBS and blocked with 3% bovine serum albumin for 30 min. Seedlings were then incubated overnight with polyclonal anti-GIK or monoclonal anti-trimethylguanosine (Calbiochem). Cy3-conjugated anti-guinea pig and FITC-conjugated anti-mouse were used as secondary antibodies. TOPRO-3 was used as a fluorescent DNA dye. Immunostaining was analyzed with a laser scanning confocal microscope (Zeiss Meta LSM510).

### In Vitro MAR Binding Assay

Recombinant proteins GIK-AT (residues 74-173) and GIK-MUT (residues 74-173; R83K) were produced in the *pQE30* expression vector carried by *E. coli* M15 cells. *ETT* MAR probes were generated by cloning SMARTest-predicted MAR sequences [60] in the *ETT* upstream genomic region into the *pCRII* vector (Invitrogen). Probe 1 (−5,233 to −5,084 bp from translation start site) and Probe 2 (−4,283 to −4,134 from translation start site) fragments were generated by EcoRI digestion and were end-labeled with a digoxigenin probe synthesis mix (Roche) using Klenow fragment (New England BioLabs). South-Western analysis was performed as described [34] with some modifications. Briefly, induced and noninduced bacterial lysates were separated by 10% SDS-PAGE and blotted onto a nitrocellulose membrane (Bio-Rad). The membrane was incubated overnight with 20 ng/mL of digoxigenin-labeled *ETT* putative MAR probes in DNA binding buffer containing 20 mM Tris-HCl, pH 7.4, 150 mM NaCl, and 20 ng/mL salmon sperm DNA at room temperature, washed, and incubated with anti-dioxigenin coupled with alkaline phosphatase (Roche). Signal was detected using CDP-Star (Roche) as a substrate. P1 and P2 probes showed similar binding efficiencies to GIK-AT. The binding result with the P2 probe is shown.

### Real-Time PCR Analysis

Total RNA was isolated from floral bud clusters at stage 10 or younger [18] using the RNeasy plant mini kit (Qiagen) and reverse-transcribed using the Superscript III RT-PCR system (Invitrogen). Quantitative real-time PCR assays were performed in triplicate with the 7900HT fast real-time PCR system (Applied Biosystems) using the SYBR Green PCR master mix (Applied Biosystems). Statistical analysis was done using paired student's *t*-test.

### ChIP Assay

The ChIP assay was performed as described [18],[72] with some modifications. Briefly, inflorescences were ground in liquid nitrogen and postfixed with 1% formaldehyde for 10 min. Chromatin was isolated and solubilized by sonication, resulting in an average DNA length of 500 bp. The solubilized chromatin was precleared with salmon sperm DNA-treated protein A- (for anti-AG, anti-dimethylated H3K9, and normal rabbit IgG) or protein G- (for anti-HA) agarose beads (Upstate). After centrifugation, the supernatant was incubated overnight with anti-AG (for AG ChIP experiments), anti-HA (Roche) (for GIK ChIP experiments), anti-modified histone (Upstate) for dimethylated H3K9, dimethylated H3K4, acetylated histone H3, and trimethylated H3K27 (for histone modification ChIP experiments), or normal rabbit IgG (for both AG and GIK ChIP experiments as a control). The DNA-protein complex was precipitated by adding protein A- or protein G-agarose beads, and the purified DNA samples were used for enrichment tests with real-time PCR assays. We measured the ratio between the input DNA before IP and bound DNA after IP for each primer set. The relative enrichment for AG and GIK ChIP experiments was the ratio obtained from: [{(B_sp_/I_sp_)/(B_ctrl_/I_ctrl_)} of Ab_sp_]/[{(B_sp_/I_sp_)/(B_ctrl_/I_ctrl_)} of control IgG], where B_sp_ = amount of bound DNA measured by a specific primer pair; I_sp_ = amount of Input DNA by a specific primer pair; B_ctrl_ = amount of bound DNA by control primer pair (*ACT*); I_ctrl_ = amount of Input DNA by control primer pair; and Ab_sp_ = anti-AG or anti-HA. The control value was set at 1.0.

The relative enrichment for the histone modification experiments was the ratio obtained from: [(B_sp_/I_sp_) of X time points]/[(B_sp_/I_sp_) of 0 h].

At least three independent biological replicates of the ChIP assay were performed for the AG ChIP and GIK ChIP experiments. Two independent biological replicates were performed for the histone modification ChIP assay. The real-time PCR assay was done in triplicate for each ChIP assay. One representative data set showing a reproducible trend is shown.

### Nuclear Matrix Isolation for Protein Analysis

Nuclei matrix was isolated as described [59] with some modifications. Briefly, nuclei were isolated using the ChIP method (see previous section) without fixation or sonication. The isolated nuclei were washed once with RSB buffer (10 mM NaCl, 3 mM MgCl_2_, 10 mM Tris-HCl, 0.5 mM PMSF, pH 7.4) and a fraction was kept as a total nuclear control. The remaining sample was digested with 50 U of DNaseI (Roche) in RSB containing 0.25 M sucrose and 1 mM CaCl_2_ for 2 h at room temperature. After centrifugation, pellets were resuspended in RSB and an equal volume of high-salt buffer I (4 M NaCl, 20 mM EDTA, 20 mM Tris-HCl, pH 7.4) and incubated for 10 min at 0°C. After centrifugation, the pellets were further extracted twice with high-salt buffer II (2 M NaCl, 20 mM EDTA, 20 mM Tris-HCl, pH 7.4, 0.25 mg/mL BSA). After high-salt extractions, the matrices were washed with RSB buffer containing 0.25 M sucrose and 0.25 mg/mL BSA and resuspended in the same buffer. The resuspended matrices and total nuclear lysates were used for western analysis. Anti-HA and anti-AG were used to detect GIK-GR-6HA and AG proteins, respectively.

### *ETT* Promoter Analysis

To generate the *pETT::GUS* construct, 8.7 kb of *ETT* upstream genomic sequence was first amplified using UltraPfu-High-Fidelity DNA polymerase with an extension time of 8 min and then cloned into the *pDONR221* (Invitrogen) to create the entry clone. Similarly, the 4.9 kb p*ETTΔMAR::GUS* construct was amplified using UltraPfu-High-Fidelity DNA polymerase with an extension time of 5 min and then cloned into the *pENTR* directional TOPO cloning vector (Invitrogen). Both clones were sequenced for confirmation. Subsequently, both entry clones were cloned into the *pBGWFS7* binary vector [69] using the Gateway cloning method. Transgenic plants with positive GUS reporter expression were crossed with *35S::GIK-GR-6HA* plants to obtain *pETT::GUS 35S::GIK-GR-6HA* and *pETTΔMAR::GUS 35S::GIK-GR-6HA* double transgenic plants. DEX treatment was performed as described above continuously at 2 d intervals. Whole inflorescences were rinsed and stained to determine GUS activity for GUS expression analysis [73].

### In Situ Hybridization

Nonradioactive in situ hybridization was performed as described [74]. Full-length *ETT* cDNA and a 3′ specific region of *GIK* cDNA were amplified with PCR and cloned into *pSK* (Stratagene) and *pCRII* vectors, respectively, and used as templates for in vitro transcription.

### Accession Numbers

*Arabidopsis* Genome Initiative locus identifiers of *Arabidopsis* genes used in this article are as follows: *AGAMOUS* (*AG*, *At4g18960*), *GIANT KILLER* (*GIK*, *At2g35270*), *ETTIN (ETT*, *At2g33860*), *CRABS CLAW* (*CRC*, *At1g69180*), *JAGGED* (*JAG*, *At1g68480*), *KNUCKLES* (*KNU*, *At5g14010*), *LEUNIG* (*LUG*, *At4g32551*), *TUBULIN 2* (*TUB*, *At5g62690*), *MU-LIKE TRANSPOSASE* (*MU*, *At4g03870*), *PHOSPHOFRUCTOSE KINASE* (*PFK*, *At4g04040*), and *LIPASE* (*At1g10740*).

## Supporting Information

Figure S1 **Semi-quantitative RT-PCR of 110 genes.** PCR products approximately 500 bp in length were amplified using primer sets designed for 110 genes located near putative AG binding sites. Primer sets were designed to span intron sequences when possible to distinguish RT-PCR products from the amplification of genomic DNA. If no amplification was detected, primers were redesigned. If after the second round of PCR no amplification was observed, the gene was considered to be a pseudogene. PCR conditions were determined using a dilution series of control DNA (2^−n^, *n* = 0–12) from 40 ng to 10 pg of genomic DNA equivalent to 4×10^5^ to 100 copies of targets per reaction. We set the conditions as follows: to one cycle of 96°C for 15 min, followed by 40 cycles of 94°C for 50 s, 60°C for 50 s and 72°C for 90 s, followed by 72°C for 10 min using Hot StartTaq DNA polymerase. In this condition, target sites in the range of 1×10^5^ and 1×10^3^ copies/reaction can be quantified, and therefore abundant genes were disregarded. *AG* and *SUP* RT-PCR products roughly correspond to 1×10^5^ and 4×10^3^ copies in our flower samples (unpublished data). After this screen, 24 genes showed reduced expression in *ag* mutant flowers, as marked by asterisks next to gene names. Left lane, 100 bp ladder (100 bp ∼1 kb in every 100 bp, 1.2 kb, and 1.5 kb; bands of 500 bp and 1 kb are thicker). Lanes show amplification products using cDNA synthesized from RNA isolated from wild-type roots, wild-type leaves, and flowers from wild-type and *ag-1* mutant plants, from left to right. Accession numbers are as follows, 1-ABP, AT1G21530; 1-ANK, AT1G04780; 1-C3H, AT1G24580; 1-CON, AT1G61740; 1-DSO, AT1G05100; 1-ENP, AT1G09060; 1-EPO, AT1G74300; 1-ERP, AT1G80690; 1-EXG, AT1G14455; 1-HLH, AT1G73830; 1-HMR, AT1G48620; 1-HYP, AT1G43690; 1-INV, AT1G56555; 1-LIP, AT1G10740; 1-PEX, AT1G14540; 1-RIG, AT1G80400; 1-SEC, AT1G56660; 1-SKK, AT1G60940; 1-SRP, AT1G47710; 1-TIN, AT1G22810; 1-TNY, AT1G74930; 1-TRA, AT1G64150; 2-AG5, AT2G42830; 2-ATH, AT2G35270; 2-BRA, AT2G19460; 2-BZP, AT2G36270; 2-CHA, AT2G02710; 2-CON, AT2G15590; 2-CTH, AT2G04240; 2-CYP, AT2G28850; 2-DOB, AT2G41940; 2-DSK, AT2G17530; 2-INI, AT2G31430; 2-LIP, AT2G15230; 2-PHD, AT2G31650; 2-RLK, AT2G02220; 2-SIG, AT2G18770; 2-SPI, AT2G39260; 2-TFL, AT2G27550; 2-TTV, AT2G31990; 2-TYK, AT2G39740; 2-WRY, AT2G37260; 2-ZIN, AT2G32930; 3-AP2, At3g54990; 3-CAK, AT3G51850; 3-EDF, AT3G58680; 3-HAT, AT3G01470; 3-HUN, AT3G21690; 3-KIN, AT3G61410; 3-KIS, AT3G44050; 3-MYB, AT3G29020; 3-PET, AT3G01350; 3-RAS, AT3G11730; 3-RBL, AT3G50330; 3-REX, AT3G06140; 3-RIN, AT3G19950; 3-SIG, AT3G53920; 3-SUN, AT3G13180; 4-AG19, AT4G22950; 4-AG21, AT4G37940; 4-AIG, AT4G09950; 4-CEL, AT4G17615; 4-CHP, AT4G02180; 4-CLC, AT4G12550; 4-GL2, AT4G17710; 4-GLU, AT4G02290; 4-GLY, AT4G02480; 4-HOX, AT4G36740; 4-MYA, AT4G12350; 4-PEC, AT4G13210; 4-PIT, AT4G09160; 4-PRG, AT4G14965; 4-PRO, AT4G10510; 4-RHF, AT4G14220; 4-RIN, AT4G09100; 4-SAB, AT4G07320; 4-SAL, AT4G39070; 4-SEN, AT4G30430; 4-SKK, AT4G11460; 4-STK, AT4G25160; 4-TOP, AT4G22360; 4-TSP, AT4G27910; 4-TUB, AT4G14960; 4-UBQ, AT4G10570; 4-UBS, AT4G10590; 5-CDC, AT5G39420; 5-CHH, AT5G57520; 5-CHR, AT5G42920; 5-CLV, AT5G62230; 5-CO, AT5G41380; 5-CYT, AT5G57570; 5-DAG, AT5G44780; 5-DIS, AT5G45500; 5-DRO, AT5G47900; 5-GAL, AT5G26920; 5-GAS, AT5G15230; 5-HAP, AT5G67180; 5-HYP, AT5G40860; 5-KIN, AT5G25440; 5-MCR, AT5G55670; 5-MYB, AT5G49330; 5-NAL, AT5G39610; 5-NAM, AT5G39540; 5-PEC, AT5G66920; 5-REK, AT5G12000; 5-RLK, AT5G35390; 5-RLL, AT5G03140; 5-SET, AT5G43990; 5-SHG, AT5G14640; 5-WRK, AT5G22570.
(1.60 MB TIF)

Figure S2 **Control ChIP assay using mock-treated** ***ag-1 35S::AG-GR*** **inflorescences.** Chromatin immunoprecipitation (ChIP) was performed using *ag-1 35S::AG-GR* inflorescences at day 0 before DEX treatments. P1, P2, and P3 indicate primer pairs used for detecting different regions of *GIK* genomic DNA. Relative enrichment was obtained from the ratio of enrichment achieved by AG antibody to that of control IgG. Enrichment of a sequence amplified from *PFK* genomic DNA was used as a basal control and was set to 1.0. Standard deviation was obtained from PCR triplicates. No significant statistical differences among the relative enrichment ratios were found.
(0.78 MB TIF)

Figure S3 **Western blotting and immunostaining using the GIK antibody.** (A) Western blotting using whole protein extracts from *Arabidopsis* leaves, roots, and flowers. Bottom panel shows Coomassie Blue staining as a protein loading control. Several larger bands were observed in roots, which may be modified GIK proteins or GIK homologs. The band in leaves was barely detectable, indicating that GIK may be regulated at the protein level. (B) Immunostaining of wild-type *Arabidopsis* root cells with anti-GIK at low magnification. Bar, 5 µm.
(1.63 MB TIF)

Figure S4 **Verification of the** ***gik*** **mutant and flower phenotypes.** (A) Isolation of homozygous *gik* plants. Homozygous plants were confirmed with PCR genotyping using primer sets P1 and P2. Plant #1 is homozygous as shown by amplification with P1 but not P2, whereas plant #2 is heterozygous as shown by amplification with both P1 and P2. All *ET14389* plants were grown on kanamycin MS-agar plates to select for the presence of the transposon before genotyping. A schematic diagram of the *GIK* coding region with the positions of the transposon insertion and the respective regions amplified by P1 and P2 are shown. (B) Expression analysis of *GIK* in the *gik* mutant using real-time PCR performed as described in Figure 4O. (C–F) *gik* mutant flowers showing bipartite anthers (* in C), a petalloid anther (D), and unfused carpels (F).
(5.21 MB TIF)

Figure S5 **Overexpression of** ***GIK*** **enhances heterozygous and homozygous backgrounds of the weak** ***ett-3*** **mutant.** (A) The gynoecium of an *ett-3/ett-3* mutant flower. (B) The gynoecium of an *ett-3/+35S:GIK-GR-6HA* flower after continuous DEX treatment. (C) The gynoecium of an *ett-3/ett-3 35S:GIK-GR-6HA* flower after continuous DEX treatment. Scale bars, 1 mm.
(1.27 MB TIF)

Figure S6 ***GIK*** **expression is not upregulated in** ***ett*** **mutant flowers.** Expression analysis of *GIK* in the *ett-1* mutant using real-time PCR with RNA extracted from the inflorescences of wild-type and *ett-1* mutant plants. Expression was normalized to the *TUB* expression. Relative expression level in the wild-type was set to 1.0.
(1.30 MB TIF)

Figure S7 **AG positively regulates the expression of** ***ETT*** **.** Time-course of *ETT* transcript expression after AG activation, as measured by real-time PCR. Inflorescences from *ag-1 35S::AG-GR* plants were treated with DEX four times at 1 d intervals and harvested at 0, 1, 2, 3, 4, 5, and 6 d after the first DEX treatment. *ETT* expression was normalized to the *TUB* RNA level. Relative expression at day 0 (0D) was set as 1.0.
(2.99 MB TIF)

Figure S8 **Endogenous GIK binds to the putative MARs of the** ***ETT*** **promoter in wild-type plants.** Wild-type inflorescences were harvested for ChIP experiments. Anti-GIK was used for immunoprecipitation. P1, P2, P3, P4, and P5 are primer pairs used to detect different regions of the *ETT* genomic DNA (as illustrated above). For details, please see the legend of Figure 5D.
(1.88 MB TIF)

Figure S9 **Effect of a deletion of distal MARs after GIK induction and real-time PCR analysis of** ***GUS*** **reporter gene expression.** (A) A plant transgenic for promoter constructs of *pETTΔMAR::GUS* with a deletion of distal MARs (* a different line from the one shown in Figure 5F) was crossed with *35S::GIK-GR-6HA* transgenic plants, and the time-course promoter analysis of the *ETT* gene after GIK induction was done as shown in Figure 5F, G. (B, C) RNA was isolated from inflorescences of *pETT::GUS 35S::GIK-GR* (B) and *pETTΔMAR::GUS 35S::GIK-GR* (C) transgenic plants shown in Figure 5E and F, respectively, at days 0, 3, and 4 after the DEX treatment. Primers specific for the *GUS* reporter gene were used for quantitative analysis. Each expression level at day 0 was set to 1.0. Paired student's *t*-test was used to analyze the differences between D0 and D3 (**p<0.01) and between D0 and D4 (**p<0.01) in (B).
(2.24 MB TIF)

Figure S10 **Analysis of histone modifications at** ***ETT*** **genomic loci.** Wild-type and *35S::GIK* inflorescences were used for the ChIP assay with antibodies for dimethylated H3K9, trimethylated H3K27, and trimethylated H3K4. Primer pairs P2, P3, and coding are shown at the top. Relative enrichment was obtained from the ratio of bound/input achieved at the respective time points to that wild-type.
(1.29 MB TIF)

Figure S11 **Real-time PCR analysis of** ***35S::GIK-GR*** **inflorescences after mock, cycloheximide (CYC), DEX, and DEX+CYC treatments.** Samples were harvested 2 h after the treatment and used for cDNA synthesis for the expression analysis of *ETT* (A), *CRC* (B), *JAG* (C), and *KNU* (D). Standard deviation was obtained from PCR triplicates.
(1.41 MB TIF)

Figure S12 **Overexpression of** ***GIK*** **affects root development.** Seedlings of Ler wild-type and *35S::GIK* plants at day 5 post-germination. Seeds of Ler wild-type and *35S::GIK* transgenic plants were planted on MS agar plates before observation of the phenotype.
(2.87 MB TIF)

Table S1 **Genes located near the putative binding sites for AG with reduced or no expression in** ***ag*** **mutant flowers.** Listed are the isolation name, accession number, position of CArG box sequences related to a gene-coding region (number of nucleotides from the initiation codon for the 5′ upstream region or from the stop codon for the 3′ downstream region), and gene description shown on the TAIR Web site (www.arabidopsis.org).
(0.03 MB DOC)

Table S2 **Sequences of oligonucleotide DNA used in this study. All are shown in the 5′ to 3′ direction.**
(0.03 MB DOC)

Table S3 **Number of T1 transgenic plants for** ***ETT*** **promoter-GUS categorized by staining strength in inflorescences.** Two lines for each construct show the results of two independent transformations.
(0.06 MB PDF)

Table S4 **List of genes tested in the time-course analysis following GIK activation.**
(0.02 MB DOC)

## Abbreviations

AG: AGAMOUS
ChIP: chromatin immunoprecipitation
CRC: CRABS CLAW
DAD1: DEFECTIVE IN ANTHER DEHISCENCE1
DEX: dexamethasone
ETT: ETTIN
GIK: GIANT KILLER
GR: glucocorticoid receptor
JAG: JAGGED
KNU: KNUCKLES
LIP: LIPASE
LUG: LEUNIG
MAR: matrix attachment region
MU: MU-LIKE TRANSPOSASE
PBS: phosphate buffered saline
PFK: PHOSPHOFRUCTOSE KINASE
RNAi: RNA interference
siRNAs: short interfering RNAs
SPL: SPOROCYTELESS
TUB: TUBULIN2
