# Supplementary figures and images for: AGAMOUS Controls GIANT KILLER, a Multifunctional Chromatin Modifier in Reproductive Organ Patterning and Differentiation
Source: PLoS Biol. 2009 Nov 24;7(11):e1000251. doi: 10.1371/journal.pbio.1000251 (PMC2774341; doi:10.1371/journal.pbio.1000251)

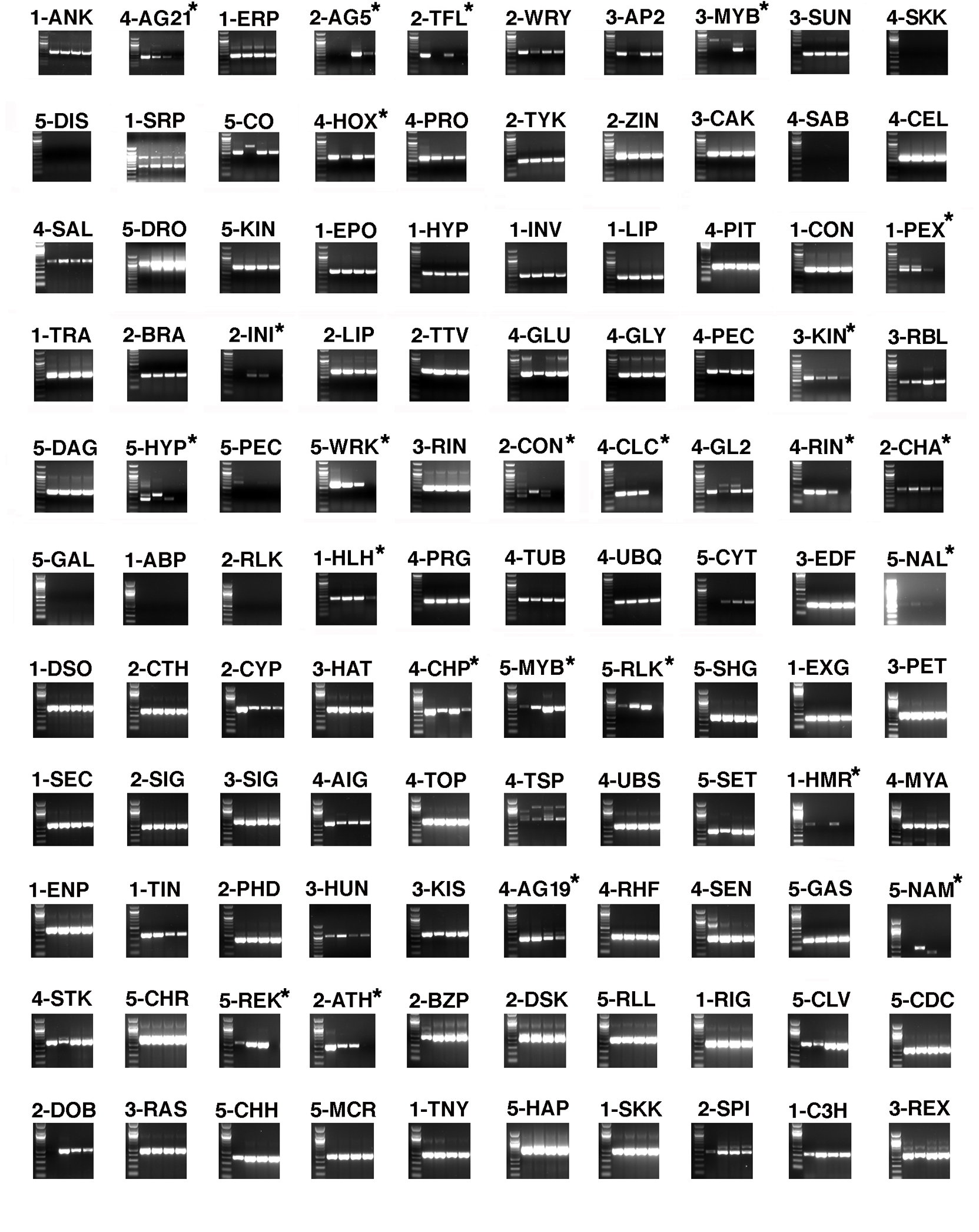

Supplement: Figure S1 — Semi-quantitative RT-PCR of 110 genes. PCR products approximately 500 bp in length were amplified using primer sets designed for 110 genes located near putative AG binding sites. Primer sets were designed to span intron sequences when possible to distinguish RT-PCR products from the amplification of genomic DNA. If no amplification was detected, primers were redesigned. If after the second round of PCR no amplification was observed, the gene was considered to be a pseudogene. PCR conditions were determined using a dilution series of control DNA (2−n, n = 0–12) from 40 ng to 10 pg of genomic DNA equivalent to 4×10^5^ to 100 copies of targets per reaction. We set the conditions as follows: to one cycle of 96°C for 15 min, followed by 40 cycles of 94°C for 50 s, 60°C for 50 s and 72°C for 90 s, followed by 72°C for 10 min using Hot StartTaq DNA polymerase. In this condition, target sites in the range of 1×10^5^ and 1×10^3^ copies/reaction can be quantified, and therefore abundant genes were disregarded. AG and SUP RT-PCR products roughly correspond to 1×10^5^ and 4×10^3^ copies in our flower samples (unpublished data). After this screen, 24 genes showed reduced expression in ag mutant flowers, as marked by asterisks next to gene names. Left lane, 100 bp ladder (100 bp ∼1 kb in every 100 bp, 1.2 kb, and 1.5 kb; bands of 500 bp and 1 kb are thicker). Lanes show amplification products using cDNA synthesized from RNA isolated from wild-type roots, wild-type leaves, and flowers from wild-type and ag-1 mutant plants, from left to right. Accession numbers are as follows, 1-ABP, AT1G21530; 1-ANK, AT1G04780; 1-C3H, AT1G24580; 1-CON, AT1G61740; 1-DSO, AT1G05100; 1-ENP, AT1G09060; 1-EPO, AT1G74300; 1-ERP, AT1G80690; 1-EXG, AT1G14455; 1-HLH, AT1G73830; 1-HMR, AT1G48620; 1-HYP, AT1G43690; 1-INV, AT1G56555; 1-LIP, AT1G10740; 1-PEX, AT1G14540; 1-RIG, AT1G80400; 1-SEC, AT1G56660; 1-SKK, AT1G60940; 1-SRP, AT1G47710; 1-TIN, AT1G22810; 1-TNY, AT1G74930; 1-TRA, AT1G64150; 2-AG5, AT2G42830; 2-ATH, A [file pbio.1000251.s001.tif]

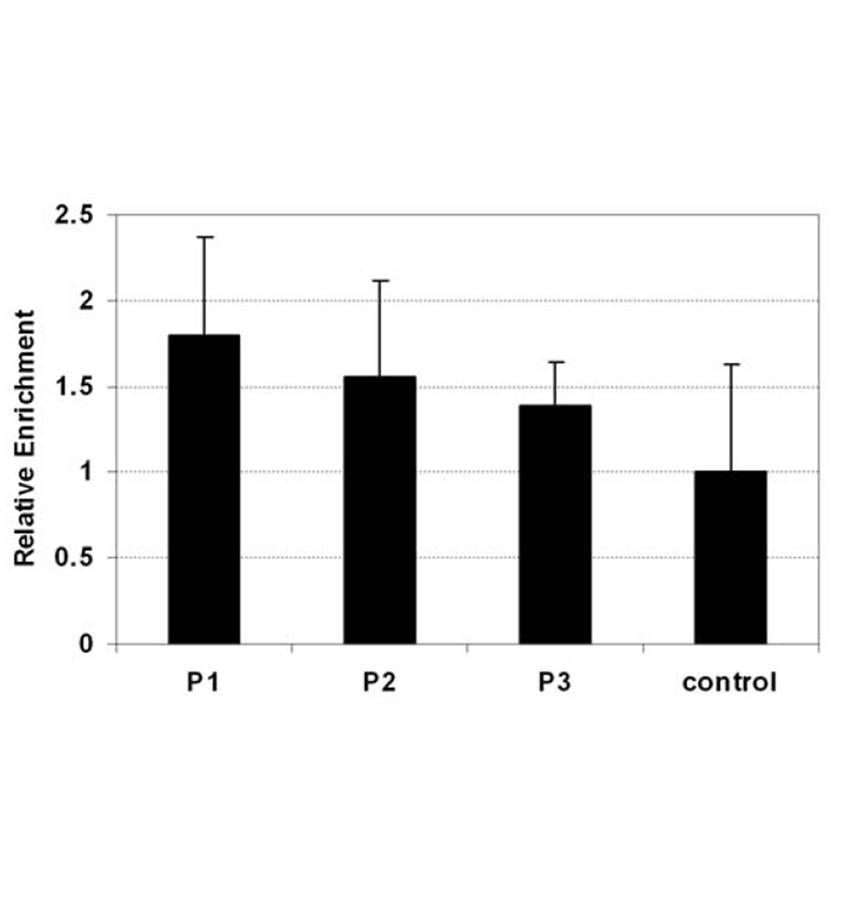

Supplement: Figure S2 — Control ChIP assay using mock-treated ag-1 35S::AG-GR inflorescences. Chromatin immunoprecipitation (ChIP) was performed using ag-1 35S::AG-GR inflorescences at day 0 before DEX treatments. P1, P2, and P3 indicate primer pairs used for detecting different regions of GIK genomic DNA. Relative enrichment was obtained from the ratio of enrichment achieved by AG antibody to that of control IgG. Enrichment of a sequence amplified from PFK genomic DNA was used as a basal control and was set to 1.0. Standard deviation was obtained from PCR triplicates. No significant statistical differences among the relative enrichment ratios were found. (0.78 MB TIF) [file pbio.1000251.s002.tif]

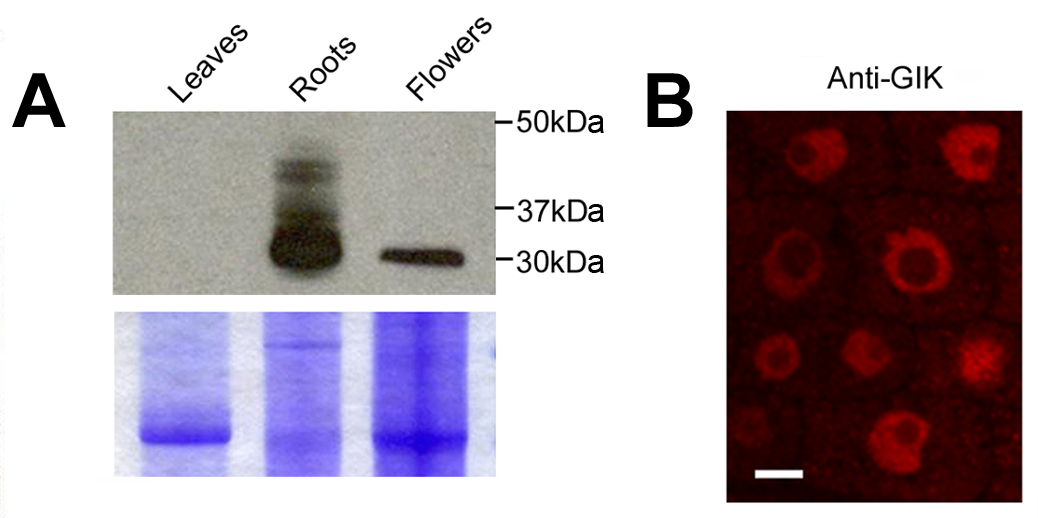

Supplement: Figure S3 — Western blotting and immunostaining using the GIK antibody. (A) Western blotting using whole protein extracts from Arabidopsis leaves, roots, and flowers. Bottom panel shows Coomassie Blue staining as a protein loading control. Several larger bands were observed in roots, which may be modified GIK proteins or GIK homologs. The band in leaves was barely detectable, indicating that GIK may be regulated at the protein level. (B) Immunostaining of wild-type Arabidopsis root cells with anti-GIK at low magnification. Bar, 5 µm. (1.63 MB TIF) [file pbio.1000251.s003.tif]

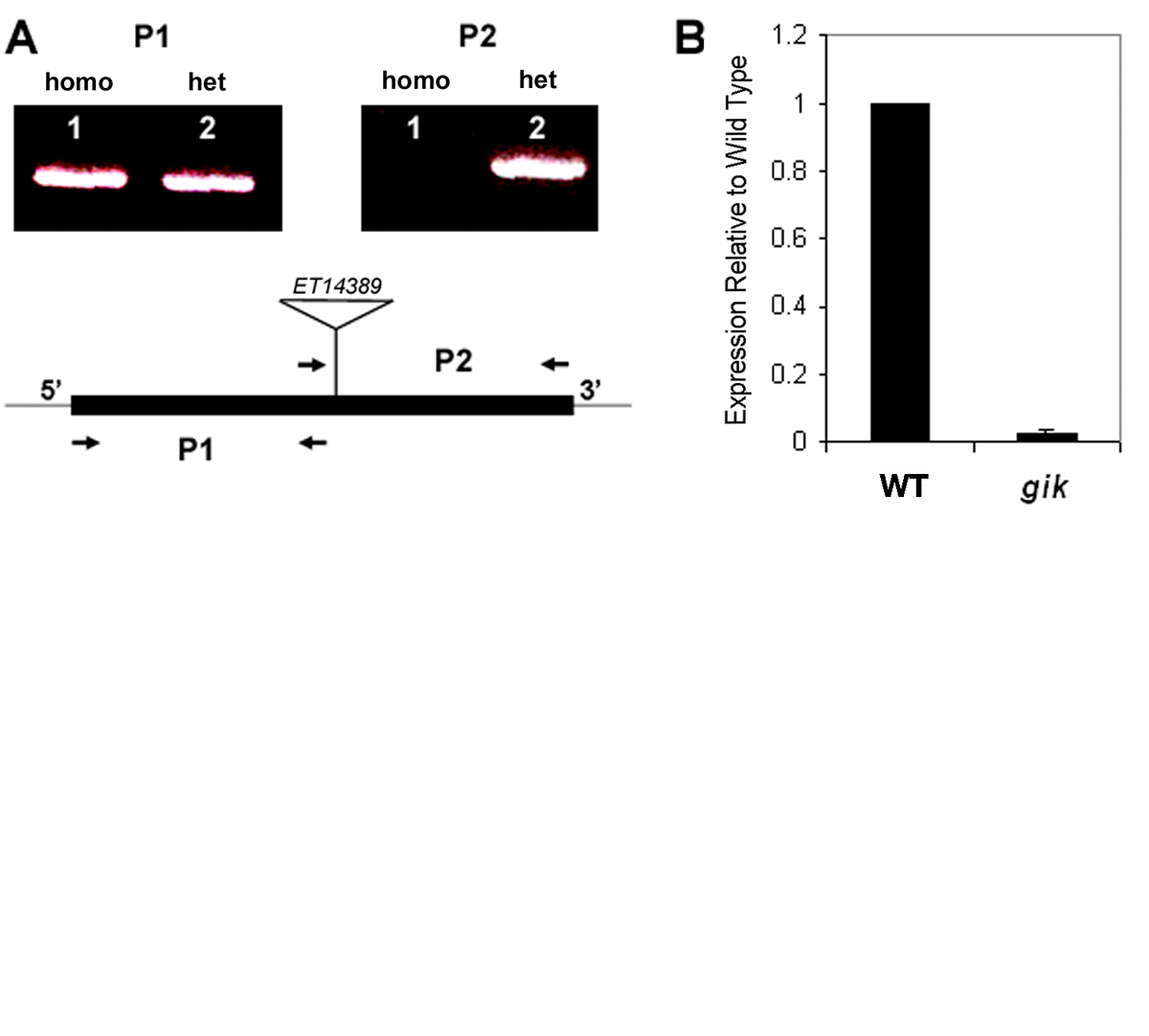

Supplement: Figure S4 — Verification of the gik mutant and flower phenotypes. (A) Isolation of homozygous gik plants. Homozygous plants were confirmed with PCR genotyping using primer sets P1 and P2. Plant #1 is homozygous as shown by amplification with P1 but not P2, whereas plant #2 is heterozygous as shown by amplification with both P1 and P2. All ET14389 plants were grown on kanamycin MS-agar plates to select for the presence of the transposon before genotyping. A schematic diagram of the GIK coding region with the positions of the transposon insertion and the respective regions amplified by P1 and P2 are shown. (B) Expression analysis of GIK in the gik mutant using real-time PCR performed as described in Figure 4O. (C–F) gik mutant flowers showing bipartite anthers (* in C), a petalloid anther (D), and unfused carpels (F). (5.21 MB TIF) [file pbio.1000251.s004.tif]

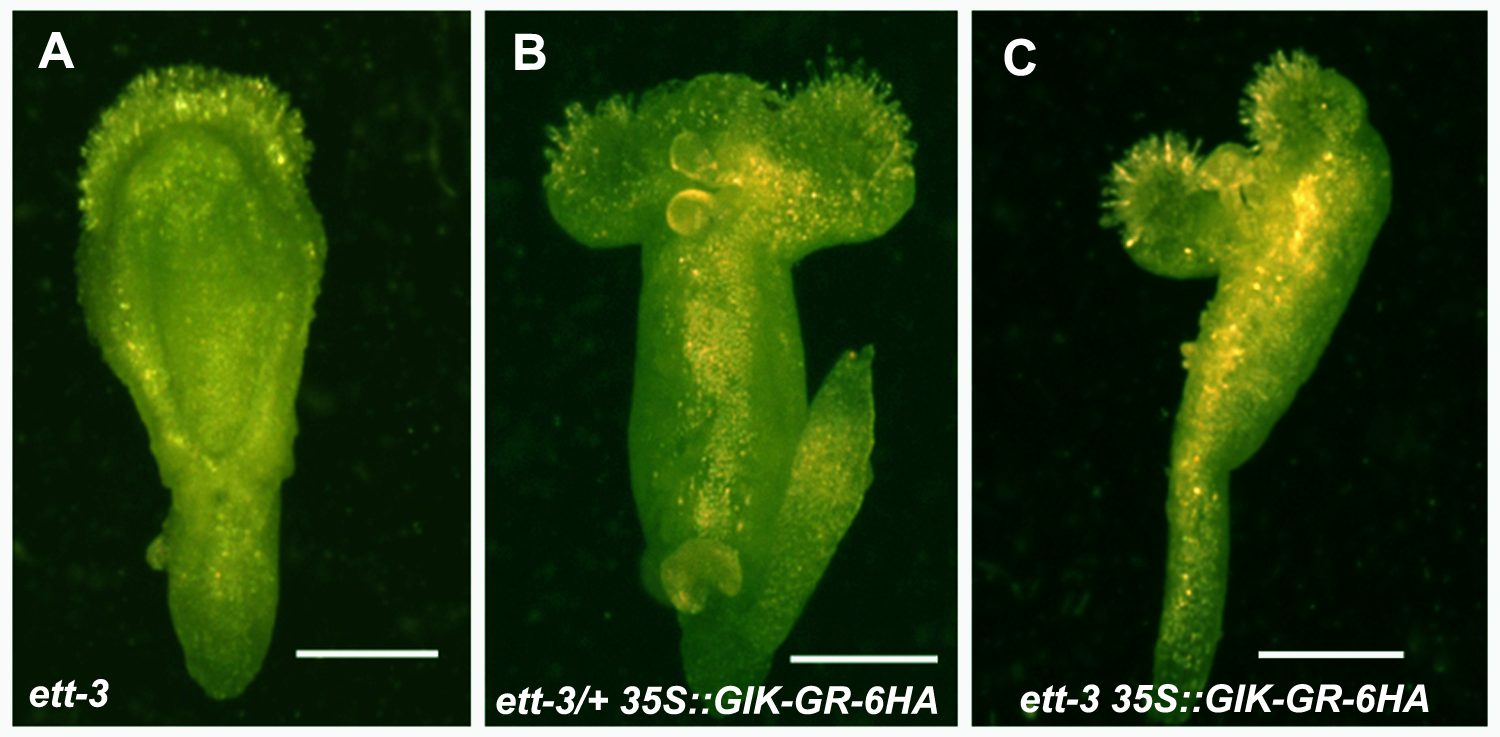

Supplement: Figure S5 — Overexpression of GIK enhances heterozygous and homozygous backgrounds of the weak ett-3 mutant. (A) The gynoecium of an ett-3/ett-3 mutant flower. (B) The gynoecium of an ett-3/+35S:GIK-GR-6HA flower after continuous DEX treatment. (C) The gynoecium of an ett-3/ett-3 35S:GIK-GR-6HA flower after continuous DEX treatment. Scale bars, 1 mm. (1.27 MB TIF) [file pbio.1000251.s005.tif]

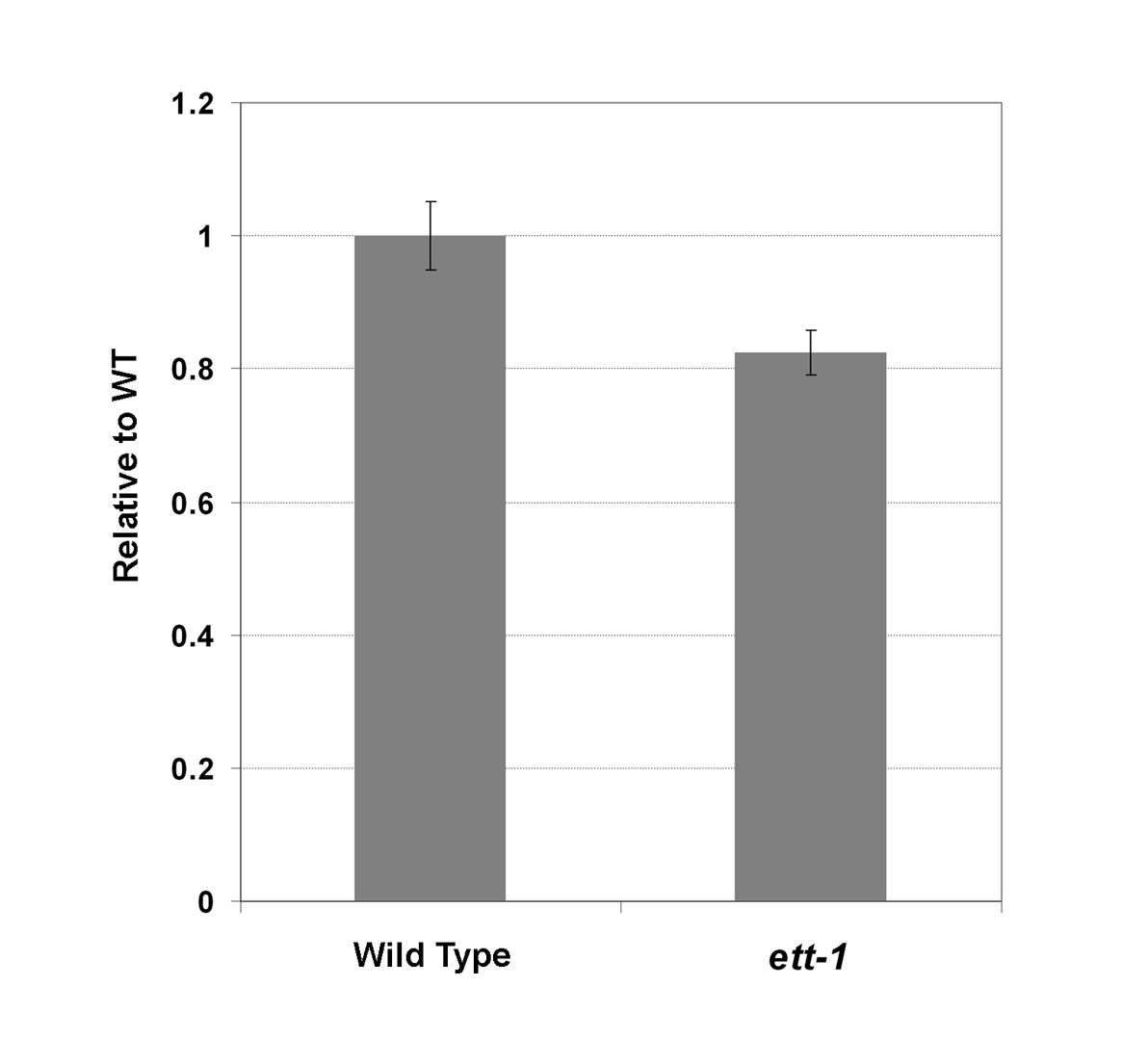

Supplement: Figure S6 — GIK expression is not upregulated in ett mutant flowers. Expression analysis of GIK in the ett-1 mutant using real-time PCR with RNA extracted from the inflorescences of wild-type and ett-1 mutant plants. Expression was normalized to the TUB expression. Relative expression level in the wild-type was set to 1.0. (1.30 MB TIF) [file pbio.1000251.s006.tif]

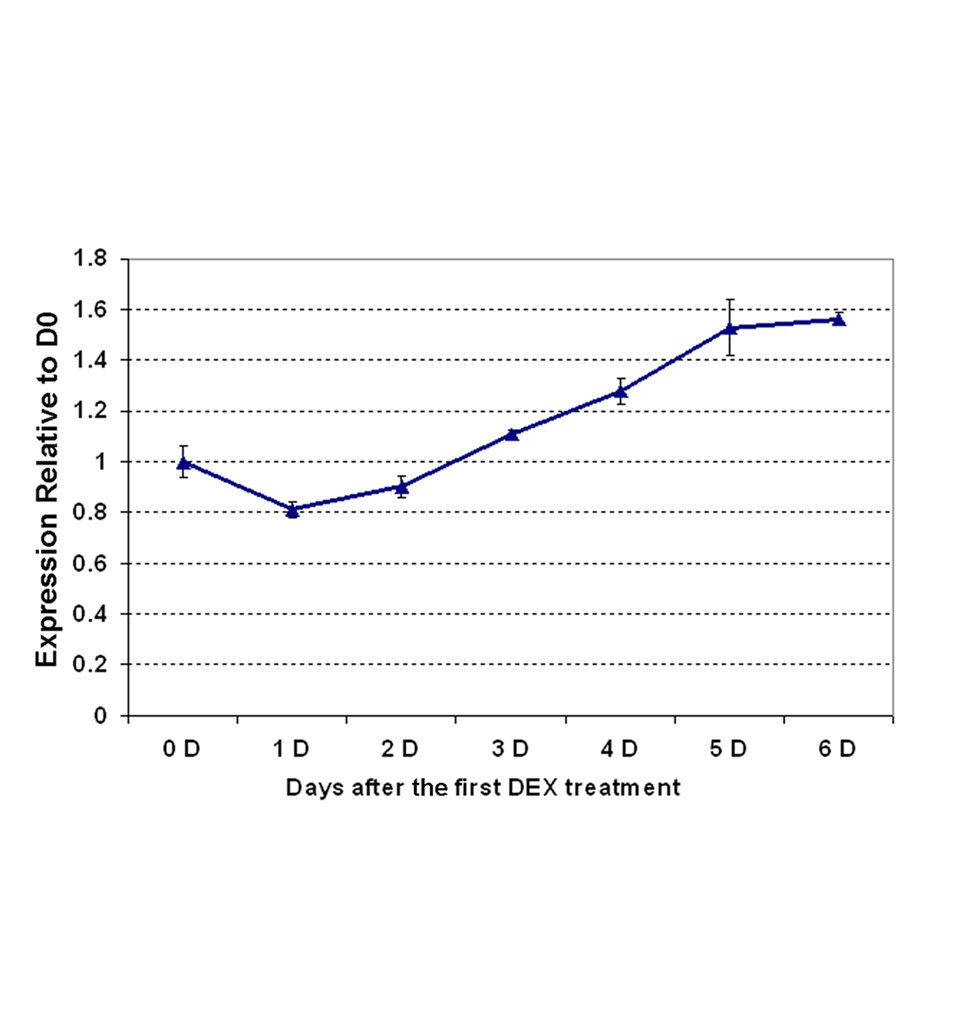

Supplement: Figure S7 — AG positively regulates the expression of ETT . Time-course of ETT transcript expression after AG activation, as measured by real-time PCR. Inflorescences from ag-1 35S::AG-GR plants were treated with DEX four times at 1 d intervals and harvested at 0, 1, 2, 3, 4, 5, and 6 d after the first DEX treatment. ETT expression was normalized to the TUB RNA level. Relative expression at day 0 (0D) was set as 1.0. (2.99 MB TIF) [file pbio.1000251.s007.tif]

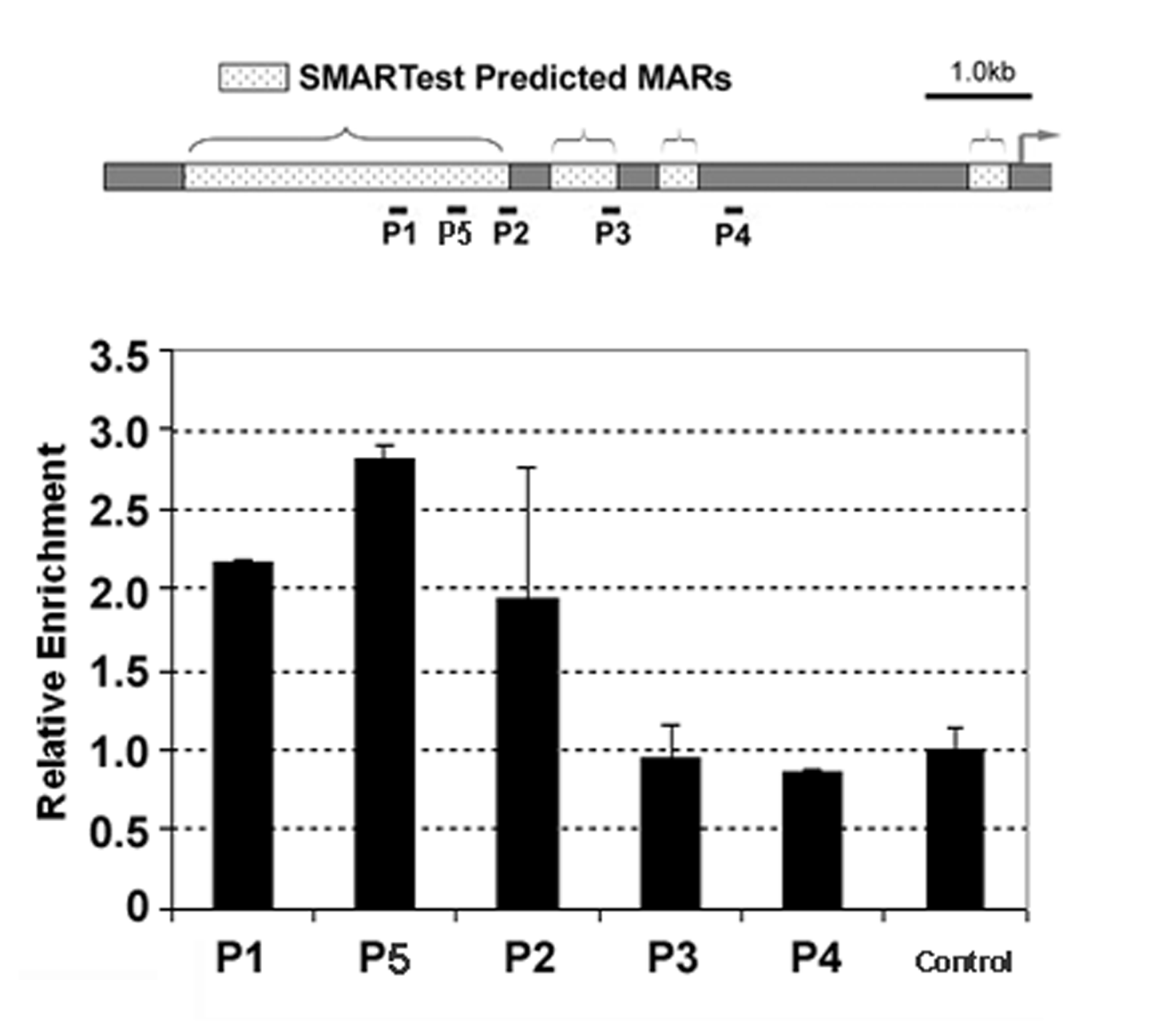

Supplement: Figure S8 — Endogenous GIK binds to the putative MARs of the ETT promoter in wild-type plants. Wild-type inflorescences were harvested for ChIP experiments. Anti-GIK was used for immunoprecipitation. P1, P2, P3, P4, and P5 are primer pairs used to detect different regions of the ETT genomic DNA (as illustrated above). For details, please see the legend of Figure 5D. (1.88 MB TIF) [file pbio.1000251.s008.tif]

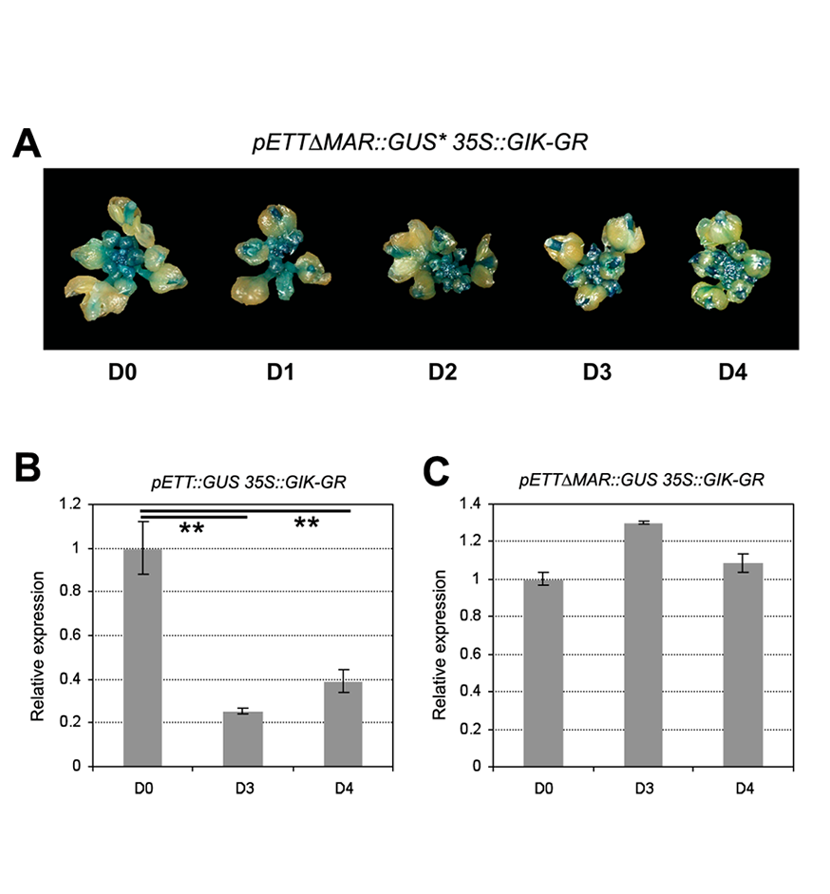

Supplement: Figure S9 — Effect of a deletion of distal MARs after GIK induction and real-time PCR analysis of GUS reporter gene expression. (A) A plant transgenic for promoter constructs of pETTΔMAR::GUS with a deletion of distal MARs (* a different line from the one shown in Figure 5F) was crossed with 35S::GIK-GR-6HA transgenic plants, and the time-course promoter analysis of the ETT gene after GIK induction was done as shown in Figure 5F, G. (B, C) RNA was isolated from inflorescences of pETT::GUS 35S::GIK-GR (B) and pETTΔMAR::GUS 35S::GIK-GR (C) transgenic plants shown in Figure 5E and F, respectively, at days 0, 3, and 4 after the DEX treatment. Primers specific for the GUS reporter gene were used for quantitative analysis. Each expression level at day 0 was set to 1.0. Paired student's t-test was used to analyze the differences between D0 and D3 (**p<0.01) and between D0 and D4 (**p<0.01) in (B). (2.24 MB TIF) [file pbio.1000251.s009.tif]

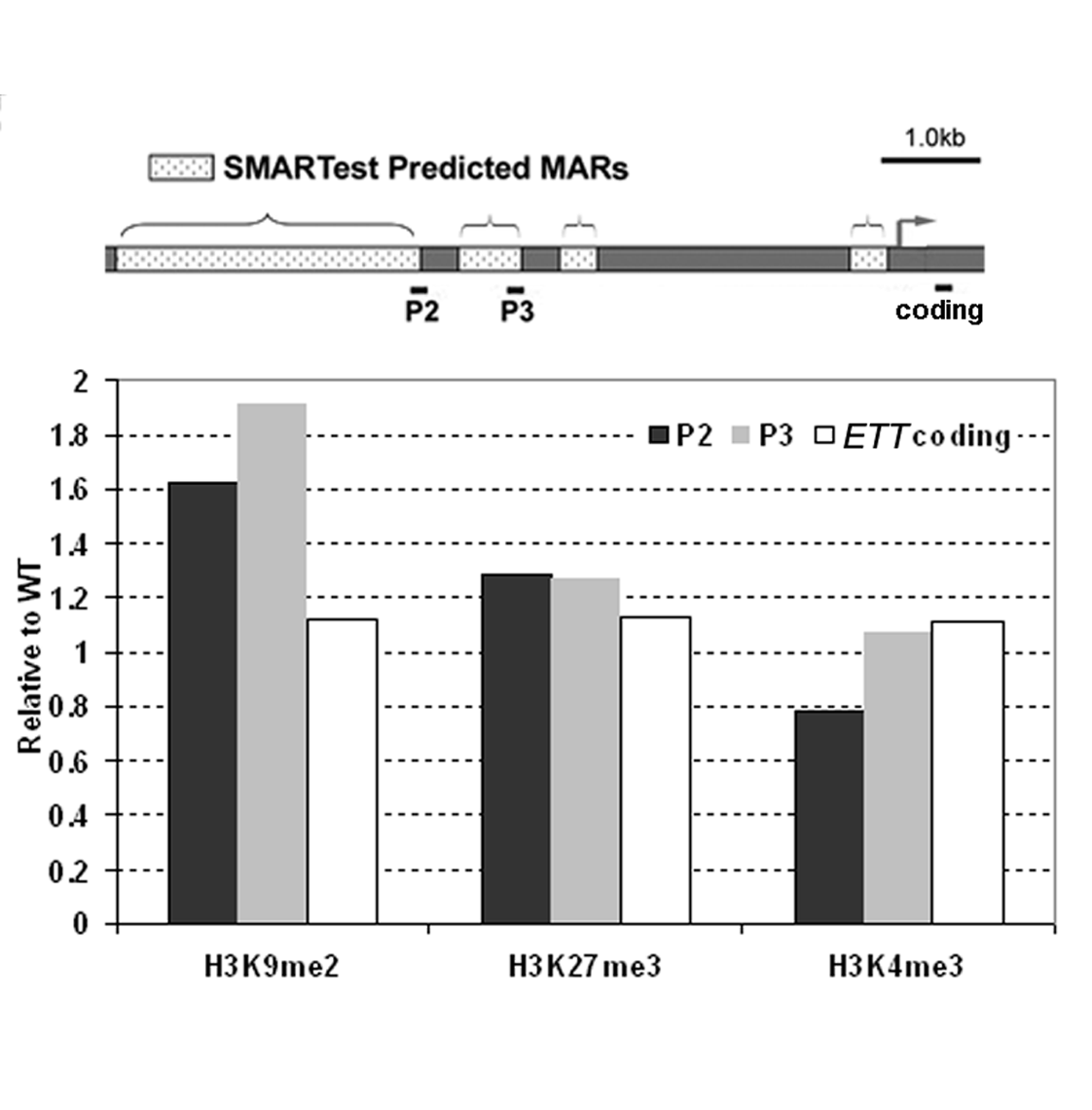

Supplement: Figure S10 — Analysis of histone modifications at ETT genomic loci. Wild-type and 35S::GIK inflorescences were used for the ChIP assay with antibodies for dimethylated H3K9, trimethylated H3K27, and trimethylated H3K4. Primer pairs P2, P3, and coding are shown at the top. Relative enrichment was obtained from the ratio of bound/input achieved at the respective time points to that wild-type. (1.29 MB TIF) [file pbio.1000251.s010.tif]

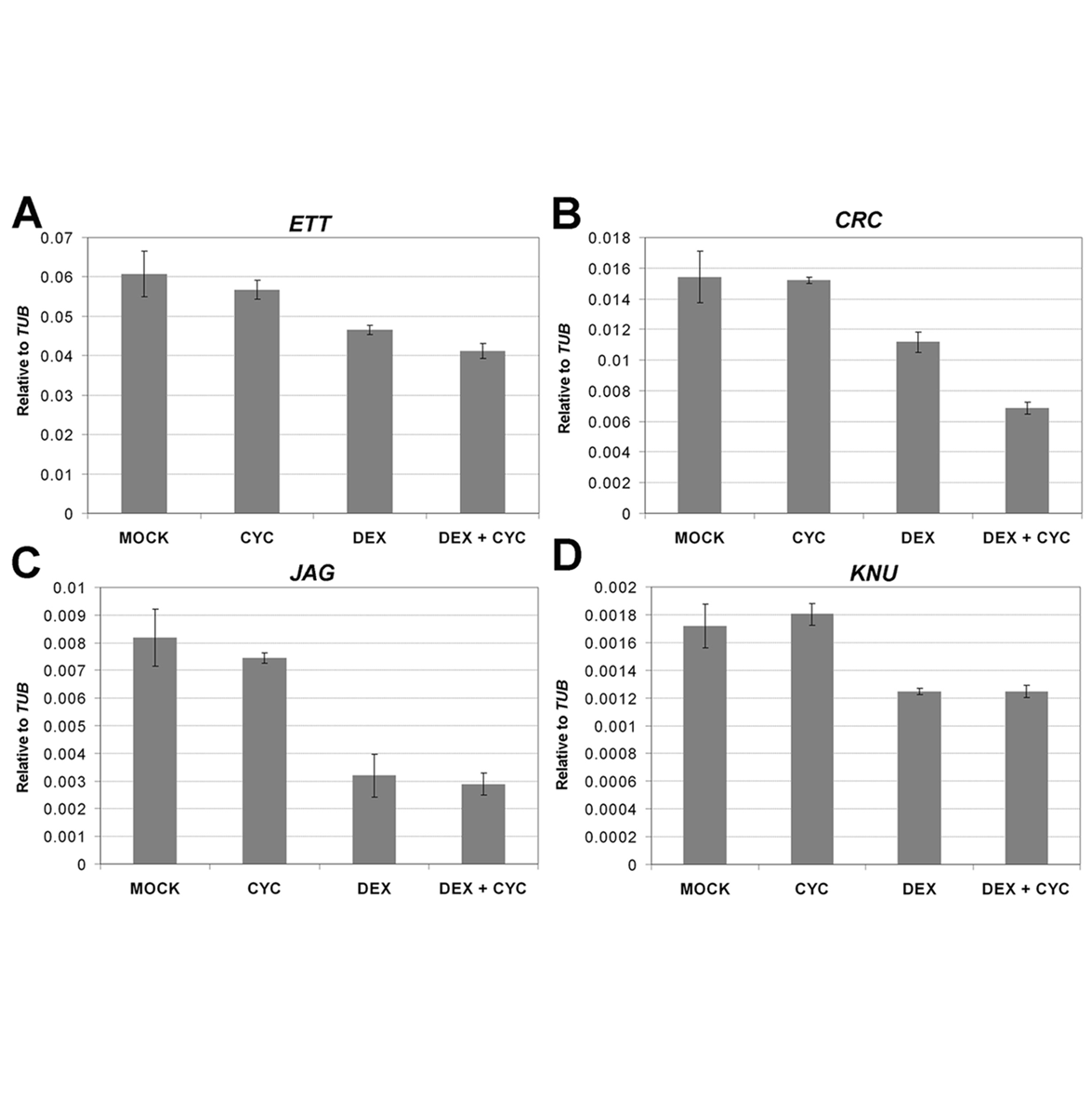

Supplement: Figure S11 — Real-time PCR analysis of 35S::GIK-GR inflorescences after mock, cycloheximide (CYC), DEX, and DEX+CYC treatments. Samples were harvested 2 h after the treatment and used for cDNA synthesis for the expression analysis of ETT (A), CRC (B), JAG (C), and KNU (D). Standard deviation was obtained from PCR triplicates. (1.41 MB TIF) [file pbio.1000251.s011.tif]

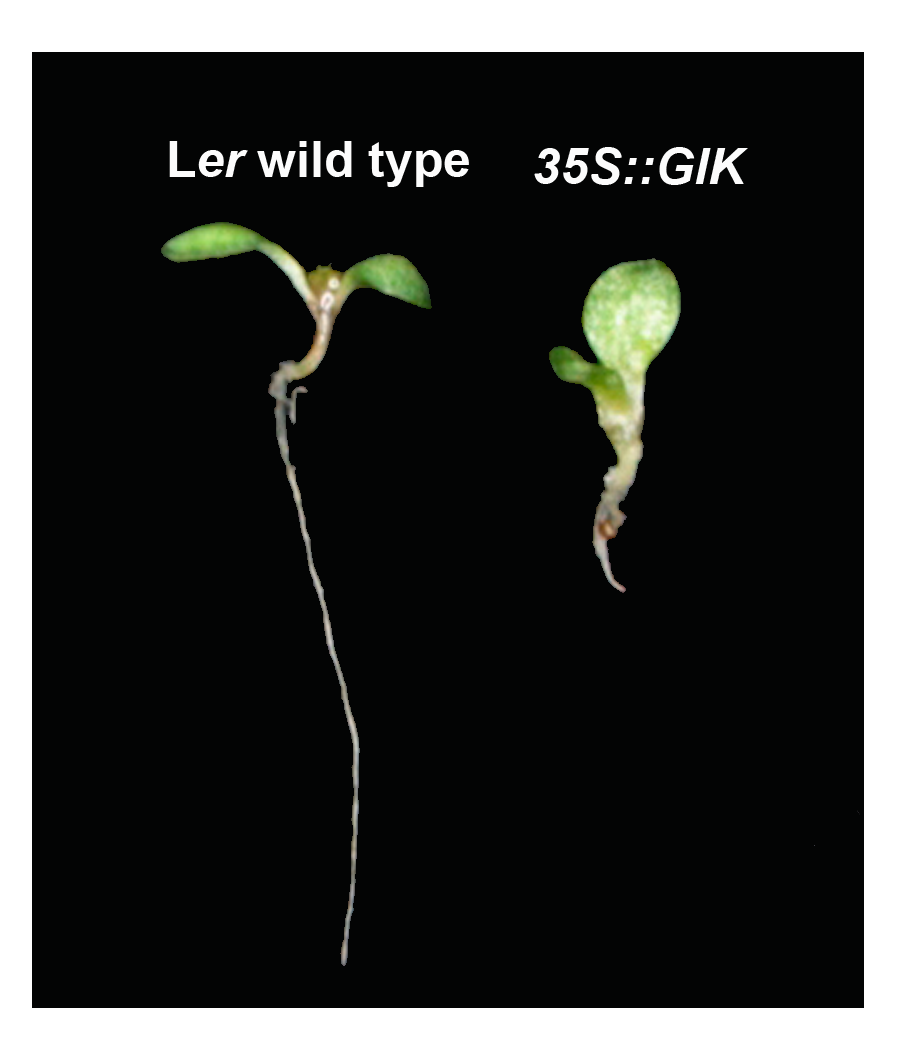

Supplement: Figure S12 — Overexpression of GIK affects root development. Seedlings of Ler wild-type and 35S::GIK plants at day 5 post-germination. Seeds of Ler wild-type and 35S::GIK transgenic plants were planted on MS agar plates before observation of the phenotype. (2.87 MB TIF) [file pbio.1000251.s012.tif]
