# Supplementary material for: AGAMOUS Controls GIANT KILLER, a Multifunctional Chromatin Modifier in Reproductive Organ Patterning and Differentiation
Source: PLoS Biol. 2009 Nov 24;7(11):e1000251. doi: 10.1371/journal.pbio.1000251 (PMC2774341; doi:10.1371/journal.pbio.1000251)
Supplement: Table S1 — Genes located near the putative binding sites for AG with reduced or no expression in ag mutant flowers. Listed are the isolation name, accession number, position of CArG box sequences related to a gene-coding region (number of nucleotides from the initiation codon for the 5′ upstream region or from the stop codon for the 3′ downstream region), and gene description shown on the TAIR Web site (www.arabidopsis.org). (0.03 MB DOC) [file pbio.1000251.s013.doc]

**Table S1. Genes Located near the Putative Binding Sites for AG with Reduced or No Expression in *ag* Mutant Flowers.**

Listed are the isolation name, accession number, position of CArG box sequences related to a gene-coding region (number of nucleotides from the initiation codon for the 5′ upstream region or from the stop codon for the 3′ downstream region), and gene description shown on the TAIR web site ([www.arabidopsis](http://www.arabidopsis/).org).

1-HLH AT1G73830 1^st^ intron bHLH protein (BEE3, BR ENHANCED EXPRESSION 3)

1-PEX AT1G14540 780 bp 5’ Anionic peroxidase (PER4)

1-HMR AT1G48620 980 bp 5’ Histone H1/H5 family member (HON5)

**2-ATH AT2G35270 730 bp 3’ AT-hook DNA binding protein (AHL21, GIK)**

2-CHA AT2G02710 460bp 5’ PAC motif-containing blue light receptor

2-CON AT2G15590 280bp 5’ Unknown protein (conserved in rice)

2-TFL AT2G27550 1.7 kb 5’ TFL1 homologue (ATC)

2-INI AT2G31430 1.3 kb 5’ Invertase/pectin methylesterase inhibitor

2-AG5 AT2G42830 750 bp 5’ AGAMOUS-LIKE5 (SHP2, SHATTERPROOF2)

3-MYB AT3G29020 130 bp 5’ AtMYB110 (myb domain protein 110)

3-KIN AT3G61410 800 bp 3’ Similar to protein kinase

4-CHP AT4G02180 920 bp 5’ DC1 domain-containing zinc finger protein

4-RIN AT4G09100 870 bp 5’ C3HC4-type RING finger protein (ATL4A)

4-CLC AT4G12550 490 bp 5’ AIR1 (Auxin-Induced in Root cultures 1)

4-AG19AT4G22950 1^st^ intron AGAMOUS-LIKR 19 (AGL19)

4-HOX AT4G36740 3.5 kb 5’ Homeodomain L-zipper protein (ATHB40)

4-AG21AT4G37940 1.0 kb 5’ AGAMOUS-LIKE 21 (AGL21)

5-REK AT5G12000 1.3 kb 5’ Similar to protein kinase

5-WRK AT5G22570 2.3 kb 5’ WRKY Transcription Factor (WRKY38)

5-RLK AT5G35390 240 bp 3’ Leucine-rich repeat protein kinase

5-NAM AT5G39540 370 bp 5’ Similar to NAC domain protein 63

5-NAL AT5G39610 3.4 kb 5’ NAC domain containing protein 92 (NAC092)

5-HYP AT5G40860 500 bp 5’ Unknown protein (conserved in rice)

5-MYB AT5G49330 2.1 kb 5’ AtMYB111 (myb domain protein 111)
