# Supplementary material for: AGAMOUS Controls GIANT KILLER, a Multifunctional Chromatin Modifier in Reproductive Organ Patterning and Differentiation
Source: PLoS Biol. 2009 Nov 24;7(11):e1000251. doi: 10.1371/journal.pbio.1000251 (PMC2774341; doi:10.1371/journal.pbio.1000251)
Supplement: Table S2 — Sequences of oligonucleotide DNA used in this study. All are shown in the 5′ to 3′ direction. (0.03 MB DOC) [file pbio.1000251.s014.doc]

**Table S2. Sequences of Oligonucleotide DNA Used in this Study**

All are shown in the 5′ to 3′ direction.

**Semi-quantitative PCR primers.**

GIK_RT_F, AGCTGGATCGAAGAACAAACCGAAGC

GIK_RT_R, TCCAATCTGAGGCATACTCATCGGC

TUB_RT_F, ATCCGTGAAGAGTACCCAGAT

TUB_RT_R, TCACCTTCTTCATCCGCAGTT

LIP_RT_F, GTGTGAGAGGTCTCGTTGATTGCC

LIP_RT_R, TTCCTGCAACGTTGGAAGATGCTGTC

**Real-time PCR primers.**

ETT_REAL_F, gtcccaagagaagcaggattggct

ETT_REAL_R, gcaagaccctctggaatctcaatg

LUG_REAL_F, atgtctcagaccaactgggaagctg

LUG_REAL_R, ccttcagcttggaaagcctgagc

CRC_REAL_F, ccacaccgtgaagctttcagtgct

CRC_REAL_R, tcaccgaatcccaagccatggatc

JAG_REAL_F, ccctatccgtggcccaagcatc

JAG_REAL_R, ccgattgatggggaacctaagagag

KNU_REAL_F, cgtcctcgctaactctccac

KNU_REAL_R, acggatgaaacggatcgtag

GIK_REAL_F, gtatctccacttacgctcgtcgg

GIK_REAL_R, GTACCCCGCAGAGTCACAACAG

TUB_REAL_F, ATCCGTGAAGAGTACCCAGAT

TUB_REAL_R, AAGAACCATGCACTCATCAGC

**ChIP primers.**

GIK_P1_f, gtatctccacttacgctcgtcgg

GIK_P1_R, GTACCCCGCAGAGTCACAACAG

GIK_P2_F, cttgcaaagtggcggcggcg

GIK_P2_R, CGGCGTGATTCCCCTGCCAAC

GIK_P3_F, GGTTGGAGGAAATTGTGCCCCTC

GIK_P3_r, CCACAATAGGCAAGTTCATTCATGGTTG

ETT_P1_F, cgatctaaatccgtcaattctaggat

ETT_P1_R, gaaaactttctactgagtaacgagtc

ETT_P2_F, gagcaatcctatacggagttcttaattac

ETT_P2_R, ggctgaatgtatgatggaatggtattgg

ETT_P3_F, caatcttagtgcggtgggtggc

ETT_P3_R, gtgcaacagaaagtaagggttggg

ETT_P4_F, ggaaacagatgtcggggcattaacc

ETT_P4_R, gatacagctatagaagagagaggcac

ETT_P5_F, Ctaatggcatacatatgtagttacttac

ETT_P5_R, ccacattgataatcaatccattagttga

CRC_P1_F, caatcgtcccatctctcacagtc

CRC_P1_R, catcctagaccagaggtgtaactg

CRC_P2_F, gacgtgtgattgatcacttgtctcc

CRC_P2_R, gaagaaaggaggagctataggatgg

CRC_P3_F, cataaacccctagatccggtccc

CRC_P3_R, cattaagctggctagggattgcaag

CRC_P4_F, gcctcctaactcataaaataagcataaccc

CRC_P4_R, ggtctttagcgaatggattgaaaaccgc

JAG_P1_F, CACTAAACAATAGCTCCCACTATC

JAG_P1_R, TACCTCCCACACTCCTTCAC

JAG_P2_F, CGTGACAGTCTTGGAGTAGTAG

JAG_P2_R, GGGGAGGTTAATGATTTGCATAAG

JAG_P3_F, CGCCGTGAAATAACGGTGTTGGG

JAG_P3_R, GGAAGCGTGCGAATCACGTGTC

JAG_P4_F, ccctatccgtggcccaagcatc

JAG_P4_R, ccgattgatggggaacctaagagag

KNU_P1_F, gcttttaatcgaagcatcatcc

KNU_P1_R, cacacacaattacagacatgttcac

KNU_P2_F, gtaggattgcttcaatttcaagc

KNU_P2_R, cttacacgatcaatcgaatctac

KNU_P3_F, agtgttcattgattcactcgtactg

KNU_P3_R, gatgggatcacagtacttctctctc

KNU_P4_F, ccttacccttgcttttaccaaaac

KNU_P4_R, ggataatgcaaaagggtacacatac

pfk_f, TGGCATCACAATTAGATTTGATCGG

pfk_r, TACAGAAGTCACACGGCTATTCGTC

mu-like_f, GATTTACAAGGAATCTGTTGGTGGT

MU-like_R, CATAACATAGGTTTAGAGCATCTGC

tub_f, ATCCGTGAAGAGTACCCAGAT

tub_R, AAGAACCATGCACTCATCAGC

ACT_F, ACTCGTTTCGCTTTCCTTAGTGTTAGCTG

ACT_R, AGCGAACGGATCTAGAGACTCACCTTG

**Cloning Primers.**

GIKG_F, GGGGACAAGTTTGTACAAAAAAGCAGGCTG

CGGCCGCAAGGTGATGAGAGTTCGATTGCTCTTGCCA

GIKG_R, GGGGACCACTTTGTACAAGAAAGCTGGGT

GCGGCCGCACGTGTGCCATCCAAGAGATTTGGACAAC

GIKRNAi_F AGCTGGATCGAAGAACAAACCGAAGC

GIKRNAi_R TCCAATCTGAGGCATACTCATCGGC

GIKRNAi1_F, ggatccccgggacgggaaccgtcact

GIKRNAi1_R, atcgatctccaatctgaggcatactcatcg

GIKRNAi2_F, Ctcgagccgggacgggaaccgtcact

GIKRNAi2_R, ggtaccctccaatctgaggcatactcatcg

GIK2RNAi1_F, GGATCCTAATCACCAGCTCCATCGTCCC

GIK2RNAi1_R, ATCGATCTGCCACGTGGACGACGACCAA

GIK2RNAi2_F, CTCGAGTAATCACCAGCTCCATCGTCCC

GIK2RNAi2_R, GGTACCCTGCCACGTGGACGACGACCAA

GIK_F, ggatccatggctggtctcgatctaggc

GIK_R, gtcgacctaaaacggagccctaccggcgcc

GIK-AT_F, ggatccgacctcgtcatgcgtcggcca

giK-AT_R, gtcgacctaaagaaaagatccggagaggga

GIK-MUT_F, ggatccgacctcgtcatgcgtcggcca

cgtggcaagccagctggatcgaag

GIK-MUT_R, gtcgacctaaagaaaagatccggagaggga

ETTG_F, GGGGACAAGTTTGTACAAAAAAGCAGGCTCGG

TGTAATGGAAATGCTACAGGATTGAATCA

ETTG_R, GGGGACCACTTTGTACAAGAAAGCTGG

GTTCCTCCGTCTCCATCACGTTCAGATCGAT

ETTGUS2_F, cacctctctcattagtcattaccattaaagtcat

ETTGUS2_R, taaagagagagaaacagagataaagataagagc

GIK_IS_F, GAGCACTTGCAAAGTGGCGGCG

GIK_IS_R, CCGGGCCCAAACGGAGCCCTACCGGCG

**Probes for MAR Binding Assay.**

ETTPROBE1_F, ctagattttaacgggacgatttat

ETTPROBE1_R, caccgtttcaatacgggttaaatc

ETTPROBE2_F, gagtttcacctgtagtataccatc

ETTPROBE2_R, gtaactacatatgtatgccattag

**Genotyping Primers.**

P1_F, atggctggtctcgatctaggc

P1_R, AGTGTTTGCGCTCTCGCGCGT

P2_F, CTTAGGGCTCACATTCTTGAAG

P2_R, aaacggagccctaccggcgcc
