# Supplementary material for: AGAMOUS Controls GIANT KILLER, a Multifunctional Chromatin Modifier in Reproductive Organ Patterning and Differentiation
Source: PLoS Biol. 2009 Nov 24;7(11):e1000251. doi: 10.1371/journal.pbio.1000251 (PMC2774341; doi:10.1371/journal.pbio.1000251)
Supplement: Table S3 — Number of T1 transgenic plants for ETT promoter-GUS categorized by staining strength in inflorescences. Two lines for each construct show the results of two independent transformations. (0.06 MB PDF) [file pbio.1000251.s015.pdf]

Table S3. Number of T1 Transgenic Plants for *ETT* Promoter-GUS Categorized by Staining Strength in Inflorescences.

Two lines for each construct show the results of two independent transformations.

	Weak	Intermediate	Strong
pETT::GUS	4	1	4
	21	4	22
pETTΔMAR::GUS	7	1	2
	41	11	24
