# Supplementary material for: AGAMOUS Controls GIANT KILLER, a Multifunctional Chromatin Modifier in Reproductive Organ Patterning and Differentiation
Source: PLoS Biol. 2009 Nov 24;7(11):e1000251. doi: 10.1371/journal.pbio.1000251 (PMC2774341; doi:10.1371/journal.pbio.1000251)
Supplement: Table S4 — List of genes tested in the time-course analysis following GIK activation. (0.02 MB DOC) [file pbio.1000251.s016.doc]

**Table S4. List of genes tested in the time-course analysis following GIK activation.**

**Gene common name Accession number Response**

***ETT* At2g33860 Down**

***CRC* At1g69180 Down**

***JAG* At1g68480 Down**

***KNU*  At5g14010 Down**

***LUG* At4g32551 No**

***NUB* At1g13400 Down**

***SPT* At4g36930 No**

***TSL* At5g20930 No**

***INO* At1g23420 No**

***STY1*  At3g51060 No**

***STY2* At4g36260 No**

***YAB2* At1g08465 No**

***YAB5* At2g26580 No**

***ARF2* At5g62000 No**

***ARF4* At5g60450 Down**

***ARF6* At1g30330 Down**

***ARF8*  At5g37020 Down**

***ARF9*  At4g23980 No**

***ARF14*  At1g35540 Up**

***WAK5*  At1g21230 Up**

***AGL8* At5g60910 No**

***RAE*  At5g06070 No**

***SUP*  At3g23130 No**

***SUP-like gene 1* At2g42410 No**

***SUP-like gene 3* At2g37740 Down**

***SUP-like gene 5* At3g23140 Up**

***SUP-like gene 6* At3g53820 No**

**Up: upregulated; Down: downregulated; No: no change or no consistent change.**
